# Neuropilin 1 and Neuropilin 2 gene invalidation or pharmacological inhibition reveals their relevance for the treatment of metastatic renal cell carcinoma

**DOI:** 10.1186/s13046-021-01832-x

**Published:** 2021-01-18

**Authors:** Aurore Dumond, Etienne Brachet, Jérôme Durivault, Valérie Vial, Anna K. Puszko, Yves Lepelletier, Christopher Montemagno, Marina Pagnuzzi-Boncompagni, Olivier Hermine, Christiane Garbay, Nathalie Lagarde, Matthieu Montes, Luc Demange, Renaud Grépin, Gilles Pagès

**Affiliations:** 1 Scientific Center of Monaco, Biomedical Department, 8 Quai Antoine Ier, MC-98000 Monaco, Principality of Monaco; 2 LIA ROPSE, Laboratoire International Associé Université Côte d’Azur - Centre Scientifique de Monaco, Nice, France; 3 Université de Paris, CiTCoM, UMR 8038 CNRS, F-75006 Paris, France; 4 Faculty of Chemistry, University of Warsaw, Pasteura 1, 02-093 Warsaw, Poland; 5 INSERM UMR 1163, Laboratory of Cellular and Molecular Basis of Normal Hematopoiesis and Hematological Disorders: Therapeutical Implications, F-75015 Paris, France; 6 Université de Paris, Imagine Institut, F-75015 Paris, France; 7 Université de Paris, LCBPT, UMR8601 CNRS, UFR Biomédicale des Saints-Pères, F-75006 Paris, France; 8 Laboratoire GBCM EA7528, Conservatoire National des Arts et Métiers, HESAM Université, 2 Rue Conté, 75003 Paris, France; 9 Université Côte d’Azur, ICN, UMR 7272 CNRS, F-06108 Nice, France; 10 University Cote d’Azur (UCA), Institute for research on cancer and aging of Nice, CNRS UMR 7284; INSERM U1081, Centre Antoine Lacassagne, Nice, France

**Keywords:** ccRCC, Neuropilins, Oncology, Immunology, Cancer

## Abstract

**Background:**

Despite the improvement of relapse-free survival mediated by anti-angiogenic drugs like sunitinib (Sutent®), or by combinations of anti-angiogenic drugs with immunotherapy, metastatic clear cell Renal Cell Carcinoma (mccRCC) remain incurable. Hence, new relevant treatments are urgently needed. The VEGFs coreceptors, Neuropilins 1, 2 (NRP1, 2) are expressed on several tumor cells including ccRCC. We analyzed the role of the VEGFs/NRPs signaling in ccRCC aggressiveness and evaluated the relevance to target this pathway.

**Methods:**

We correlated the NRP1, 2 levels to patients’ survival using online available data base. Human and mouse ccRCC cells were knocked-out for the *NRP1* and *NRP2* genes by a CRISPR/Cas9 method. The number of metabolically active cells was evaluated by XTT assays. Migration ability was determined by wound closure experiments and invasion ability by using Boyden chamber coated with collagen. Production of VEGFA and VEGFC was evaluated by ELISA. Experimental ccRCC were generated in immuno-competent/deficient mice. The effects of a competitive inhibitor of NRP1, 2, NRPa-308, was tested in vitro and in vivo with the above-mentioned tests and on experimental ccRCC. NRPa-308 docking was performed on both NRPs.

**Results:**

Knock-out of the *NRP1* and *NRP2* genes inhibited cell metabolism and migration and stimulated the expression of VEGFA or VEGFC, respectively. NRPa-308 presented a higher affinity for NRP2 than for NRP1. It decreased cell metabolism and migration/invasion more efficiently than sunitinib and the commercially available NRP inhibitor EG00229. NRPa-308 presented a robust inhibition of experimental ccRCC growth in immunocompetent and immunodeficient mice. Such inhibition was associated with decreased expression of several pro-tumoral factors. Analysis of the TCGA database showed that the NRP2 pathway, more than the NRP1 pathway correlates with tumor aggressiveness only in metastatic patients.

**Conclusions:**

Our study strongly suggests that inhibiting NRPs is a relevant treatment for mccRCC patients in therapeutic impasses and NRPa-308 represents a relevant hit.

**Supplementary Information:**

The online version contains supplementary material available at 10.1186/s13046-021-01832-x.

## Background

Clear cell Renal Cell Carcinoma (ccRCC) is the most common form of kidney cancers [1, 2]. If non-metastatic, surgery remains the best treatment. However, metastatic ccRCC (mccRCC) are chemo- and radio-resistant. Most of ccRCC are inactivated for the *von Hippel-Lindau* gene (*VHL*), leading to Hypoxia Inducible Factor-1 and 2 alpha (HIF-1, 2α) stability [3]. HIFs are key players in tumor aggressiveness and drug resistance particularly through Vascular Endothelial Growth Factor-A (VEGFA)-dependent angiogenesis and VEGFC-dependent lymphangiogenesis [3].

VEGFA exerts its activity through its receptors VEGFR1 and − 2 and its coreceptor Neuropilin 1 (NRP1) and VEGFC through VEGFR2 and − 3 and Neuropilin 2 (NRP2) [4].

Thus, the current reference treatments target the VEGFs/VEGFRs dependent angiogenesis. Two strategies are currently used: i) antibodies or decoy receptors targeting VEGFA such as bevacizumab (Avastin®) or aflibercept (Zaltrap®) and ii) tyrosine-kinase inhibitors (TKi) targeting the kinase domain of VEGFs receptors such as sunitinib (Sutent®) [5].

The efficacy of the current first-line treatment sunitinib varies from a patient to another with a median survival varying from few months to few years [6]. This transient effect is in part explained by the development of an alternative lymphatic network dependent on the production of VEGFC by tumor cells or cells of the microenvironment in reaction to the stress induced by the drug [7]. Hence, the destruction of the vascular network leads to the development of an alternative network favoring metastatic dissemination. These results highlight the need to develop alternative therapeutic strategies for the treatment of mccRCC especially at relapse on anti-angiogenic drugs.

To prevent redundancy of the vascular and lymphatic networks in response to the reference treatments, the VEGFA/VEGFRs coreceptors, the NRPs, may represent relevant targets in oncology [8–10]. NRPs, 120–130 kDa transmembrane glycoproteins, were initially described as mediators of neuronal guidance. NRP1 and NRP2 share 44% amino acid sequence identity and close domain structures. They form ternary complexes with VEGFs and their tyrosine kinase associated domains and represent key actors of the pro-angiogenic and pro-lymphangiogenic signaling pathways. NRPs are also expressed on immune cells where they exert an activation or a repression of the immune response [11]. Moreover, NRPs overexpression in cancer cells is correlated to a high metastatic potential and to a poor prognosis [12]. Down-regulation of *NRP1* by shRNA in ccRCC cells decreases migration, invasion and experimental human tumor growth [10], while *NRP2* down-regulation results in decreased tumor cell extravasation in the lymphatic network and reduced cell metastatic dissemination in immunodeficient models [9]. Thus, targeting NRPs in ccRCC appears as a relevant therapeutic strategy. To this end, we developed a NRPs inhibitor, NRPa-308. It exerts anti-angiogenic and anti-proliferative effects, and prevents the growth of experimental models of highly aggressive triple negative breast cancers [8, 13].

The aim of this study was to validate the relevance of NRPs targeting in models of ccRCC generated in the presence of an active immune system. Although immunotherapy showed promising results in mccRCC, only 30% of patients beneficiate of the treatment [14]. We further determined the antitumor effect of NRPa-308 on experimental ccRCC and compared its efficacy to the referent treatment sunitinib through in vitro and in vivo approaches.

## Materials and methods

### Reagents

NRPa-308 has been synthesized at the University of Paris (Luc Demange’s team). Sunitinib was purchased from Selleckem or from residual materials given to patients (Center Antoine Lacassagne, Nice, France) and prepared as a 2.5 mmol/L stock solution in dimethyl sulfoxide (Sigma, 472,301) and stored at − 20 °C. EG00229 was purchased from Tocris.

### Cell lines

786-O, A498 and RENCA cell lines were purchased from the American Tissue Culture Collection (ATCC). They were cultured as indicated by ATCC and as already described [15].

### Genomic disruption of Neuropilins using CRISPR-CAS9 and shRNA

786-O or RENCA cells were transfected with PX458 plasmids containing CRISPR-Cas9 targeting region of the second exon of the *NRP1* or of the *NRP2* gene using NaCl and PEI. Control cells were obtained by transfecting the empty plasmid. The sgRNA sequence used to target the human *NRP1* gene was: 5′-CGGGTACCTTACATCTCCTG-3′; the sgRNA sequence targeting the human *NRP2* gene was: 5′- TTCAAACGACCTCCGCACGG-3′. The sgRNA sequence used to target the mouse *nrp1* gene was: 5′- GCAAGACTCGAATCCTCCCGG − 3′; the sgRNA sequence targeting the mouse *nrp2* gene was: 5′- GTGGATCAAATAGTAACGTG − 3′. As the PX458 plasmid contains GFP, cells were first sorted using flow cytometry to obtain cells containing the CRISPR-Cas9 and followed by clonal selection and screening. Sequencing of human genomic DNA to confirm the mutations leading to human *NRP1* or *NRP2* invalidation was performed using the following primers: Forward *NRP1* 5′- CACGAAGGACTTACGGGG-3′ and Reverse *NRP1* 5′- AGACAGGCGTGACCAGTAG-3′, and Forward *NRP2* 5′- TGAGCCGGAATAATCTCTTCCAC-3′ and Reverse *NRP2* 5′- GGTGCTTACTTGCAGTCGTG-3′. Sequencing of mouse genomic DNA was performed with the following oligonucleotides: Forward *nrp1* 5′- ACAGGGCCCGAATGTTCTC − 3′ and Reverse *nrp1* 5′- TTCACAGACTCCATTGCCTG − 3′, and Forward *nrp2* 5′- TTTACATCAAGGCATTGGCAG − 3′ and Reverse *nrp2* 5′- GTGGAAGTTACGGGATCGTATAGTC − 3′.

### Quantitative real-time PCR (qPCR) experiments

One microgram of total RNA was used for the reverse transcription, using the QuantiTect Reverse Transcription Kit (Qiagen), with blend of oligo (dT) and random primers to prime first strand synthesis. SYBR Master Mix Plus (Eurogentec) was used for quantitative real-time PCR (qPCR). The mRNA level was normalized to 36B4 mRNA. For oligo sequences, see Table S1.

### Protein level measurement by flow cytometry analysis (FACS)

Knocked-out (KO) cells were incubated for one hour with primary antibodies: i) Polyclonal sheep IgG Human anti-NRP1 antibody (AF3870 (sheep); R&D systems); ii) Polyclonal goat IgG Human/Mouse/Rat anti-NRP2 antibody (AF2215 (goat); R&D systems). After washing with cold PBS, the cells were incubated for 30 min with the secondary antibodies: i) Donkey Anti-Sheep IgG H&L, Alexa Fluor 594 (Abcam); ii) Goat anti-Human IgG (H + L) Cross-Adsorbed Secondary Antibody, Alexa Fluor 488 (Thermo Fisher). The cells were then washed with cold PBS and resuspended in the FACS medium (cold PBS + 2.5 mM EDTA). NRP1 and NRP2 level expression were determined using a fluorescence-activated cell sorter (FACS Melody BD Biosciences) with a 488 nm and a 594 nm laser beam.

### Measurement of cytokines

CXCL8 cytokines and VEGFA were detected by using PeproTech ELISA kits according to the manufacturer’s indication as already described [16]. VEGFC and CXCL5 were measured using R&D systems ELISA kits according to the manufacturer recommendations.

### Measurement of cell migration velocity and invasive ability

At confluency, a wound was created on the cell monolayer and its width was measured every hour for 10 h to determine the migration velocity. At the end of the experiment, the cells were counted to verify if cell death or proliferation had not influenced the wound closure. To evaluate invasion, cells were seeded onto the upper side of the collagen-coated filters of Boyden chambers containing polycarbonate membranes (8-μm pores, Transwell; Corning, Sigma). Cell invasion was followed for 24 h at 37 °C in 5% CO2. Invasive cells on the lower membrane surface were fixed in 3% paraformaldehyde, stained with 0.4% crystal violet. Cells were counted using the Image J software.

### Competitive NRP1/2 VEGFA/VEGFC binding assay

The flat bottom surface of a 96-well plate was coated with 100 μL (200 ng/well) recombinant human NRP1 or NRP2 and incubated overnight at 4 °C. Non-specific binding was blocked by the incubation with 0.5% BSA in PBS. 50 μL of NRPa-308 dissolved in range concentrations and 50 μL (400 ng/mL) of human (bt)-VEGFA or VEGFC in PBS containing 4 μg/mL of heparin were mixed. After two hours incubation at room temperature, the (bt)-VEGFA plate was washed and treated with streptavidin-horseradish peroxidase (HRP) conjugate in PBS (1:8000). The VEGFC plate was incubated with (bt)-anti-VEGFC for one hour and then revealed using HRP conjugate. Luminescence was quantified immediately after addition of 100 μL chemiluminescent substrate. In a positive control, only (bt)-VEGFA or VEGFC was present in wells, while, in negative control (NS), wells were not coated with NRP1 or NRP2. Percentages of inhibition were calculated by the following formula: 100% − [[(S − NS)/(P − NS)]∙100%], where S is the signal intensity measured, NS is the signal measured in negative control, and P is the signal measured in positive control. Presented data are the mean ± SEM of two or three independent experiments, each performed in triplicate.

### Docking study

NRP1 (PDB ID: 6FMF) and NRP2 (PDB ID: 5DN2) structures were retrieved from the Protein Data Bank (PDB) [17]. The NRP2 structure was aligned with the NRP1 structure and both structures were prepared using MGL tools (https://www.ncbi.nlm.nih.gov/pubmed/19399780. (Accessed: 1st February 2019). Three-dimensional conformations of NRPa-308 were generated using iCon, the LigandScout v.4.3 conformer generator [18] (defaults settings of the BEST option were used, except for the maximum number of conformations generated that was set to 50 instead of 25). Protein – ligand docking of compound NRPa-308 into the NRP1 and NRP2 structures was performed using AutoDock Vina v.1.1.2 [19]. The x, y, z grid centre coordinates used are 12.045, 21.518, 15.783 and the size of the search space was set to 20 Å × 20 Å × 20 Å. Only the pose associated with the best score was considered for each run.

### Tumor xenograft formation, size evaluation and treatment

786-O cells expressing luciferase (Luc 1) or RENCA cells expressing luciferase (Luc 2) were injected subcutaneously into the flanks of 5 weeks old nude female mice or Balb-C mice. Treatment by NRPa-308 in carboxymethyl cellulose was carried out by oral gavage 3 days a week; the control group was treated with carboxymethyl cellulose. Tumors measurements were carried out once a week with a caliper and by luciferase measurements with IVIS chamber as previously described [20]. All animal procedures were performed according to the Monaco animal experimentation guidelines in strict accordance with the recommendations in the Guide for the Care and Use of Laboratory Animals. Our experiments were approved by our internal ethic committee.

### Immuno-fluorescence

Tumor sections (5 μm cryostat sections) were fixed in 4% paraformaldehyde for 20 min at room temperature and blocked in 1% donkey serum in tris-buffered saline (TBS) for 2 h. Sections were then incubated overnight with anti-rabbit LYVE-1 polyclonal (Ab14917, 1:200; Abcam) or rat monoclonal anti-mouse CD31 (clone MEC 13.3, 1:1000; BD Pharmingen) and monoclonal anti- mouse α-smooth muscle actin (αSMA A2547, 1:1000; Sigma) antibodies. Preparations were mounted and analyzed with a Leica microscope, and counted at a 10x magnification.

### Patients online data

Normalized RNA sequencing (RNA-Seq) data of The Cancer Genome Atlas (TCGA) were downloaded from cBioportal (www.cbioportal.org, TCGA Provisional; RNA-Seq V2). Data were available for 534 RCC tumor samples or from 1020 different cell lines. The results published here are in whole or in part based upon data generated by the TCGA Research Network: http://cancergenome.nih.gov/ [21, 22].

### Statistical analysis

Statistical significance and *P* values were determined with the two-tailed t-test. The Kaplan–Meier method was used to produce survival curves and analyses of censored data were performed using the log-rank test.

## Results

### *NRP1* or *NRP2* gene invalidation resulted in inhibition of cell proliferation and migration

**Fig. 1:**
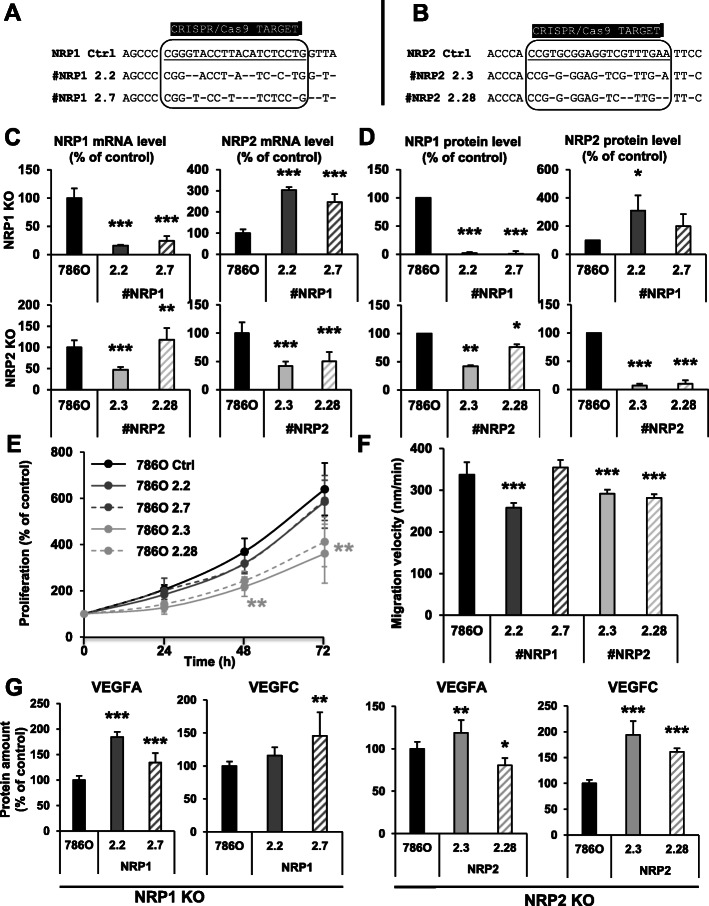
*NRP1* or *NRP2* gene invalidation results in inhibition of cell proliferation and migration. **a** The locus of the *NRP1* gene was sequenced in control (NRP1 Ctrl) and in two independent clones (#NRP1 2.2 and #NRP1 2.7) KO for *NRP1*. **b** The locus of *NRP2* was sequenced in control (NRP2 Ctrl) and in two independent clones (#NRP2 2.3 and #NRP1 2.28) KO for *NRP2*. **c** NRP1 and NRP2 mRNA levels were tested by qPCR in control (786O), in two independent clones (#NRP1 2.2 and 2.7) KO for *NRP1*, and in two independent clones (#NRP2 2.3 and 2.28) KO for *NRP2.* **d** NRP1 and NRP2 protein levels were evaluated by flow cytometry in control (786O), in two independent clones (#NRP1 2.2 and 2.7) KO for *NRP1*, and in two independent clones (#NRP2 2.3 and 2.28) KO for *NRP2.* **e** The proliferation of *NRP1* and *NRP2* KO cells were tested by counting the cells at the indicated time points. **f** The migration of *NRPs* KO cells was determined in scratch assays by measuring the time of wound closure. **g** VEGFA and VEGFC expression was tested in control (Ctrl) and KO clones by ELISA. *p < 0.05; **p < 0.01; *** *p* < 0.001

According to the papers of Cao Y et al [9, 10], neither *NRP1* or *NRP2* knock-down impacted cell proliferation (*NRP1*/*NRP2*) and migration (*NRP2*). These results were surprising since they described that *NRP1* knock-down decreased the AKT activity, a major pathway involved in cell proliferation/survival. Moreover, the NRPs-mediated signaling pathways were associated with cell proliferation and migration in several cancers [23]. We first tried to confirm the results of Cao Y et al particularly on cell proliferation and migration by using the same shRNA and two other independent shRNA through lentiviral infection. The knock-down levels that we obtained were comparable to those described by Cao Y et al (Fig. S1A-B). *NRP1* knock-down decreased *NRP2* expression, an observation that was not described in the Cao Y et al papers. This observation, confirmed with two independent shRNA, eliminates an unspecific effect, and suggests a crosstalk between the NRP1 and NRP2 signaling pathways (Fig. S1A-B). Whereas Cao Y et al did not detect modifications of cell proliferation and migration at 24 h, we observed a small but significant inhibition of cell metabolic activity for the shNRP1 cells at 72 h and a surprising increased cell metabolic activity for the shNRP2 cells (Fig. S1C). The migration velocity was also significantly inhibited for shNRP1 and shNRP2 cells (Fig. S1D). The privileged NRP1 ligand VEGFA and the privileged NRP2 ligand VEGFC were not affected by the knockdowns (Fig. S1E). These differences with the results of Cao Y et al incited us to decipher the role of NRP1 and NRP2 by knocking-out (KO) their genes by the CRISPR/Cas9 method in human (786-O) and mouse (RENCA) ccRCC cells. Two independent KO clones for *NRP1* and *NRP2* genes were obtained for 786-O (Fig. 1a-b), and one KO clone for *NRP1* and *NRP2* genes for RENCA (Fig. S2A) cells. Specific NRP1 and NRP2 mRNA levels were very low and protein levels were almost undetectable in the KO clones (Fig. 1c-d and Fig. S2A). However, *NRP1* KO tends to increase NRP2 levels whereas *NRP2* KO tends to decrease NRP1 levels in 786-O cells at the mRNA and protein levels (Fig. 1d). A completely different guide RNAs for *NRP1* and *NRP2* [24] genes that were positioned adequately for the presence of the PAM sequence [25] excluded also unspecific effects. Although the trend in decreased NRP1 levels were consistently observed in *NRP2* KO RENCA cells, the *NRP1* KO resulted in decreased expression of NRP2 in RENCA cells (Fig. S2A). We obtained opposite results between KO and down-regulation of NRP1 by shRNA for NRP2 expression in 786-O cells (NRP1-directed shRNA decreased NRP2 levels whereas the KO tends to induce NRP2 expression). All these results, recapitulated in Tables S2, were consistent in 786-O and RENCA cells with down-regulation or KO. They suggest a fine-tuned crosstalk between the NRP1 and the NRP2 signaling, which mediates an equilibrated expression of each protein compatible with cell metabolic activity/survival. In 786-O cells, *NRP1* KO moderately impacted cell metabolic activity while *NRP2* KO decreased it more importantly (Fig. 1e). These results were consistent for NRP1 down-regulation and KO whereas down-regulation of NRP2 stimulated and KO inhibited cell metabolic activity (Fig. 1e and Fig. S1D). A moderate but still non-significant inhibition of cell metabolic activity was also observed in RENCA *NRP1* KO cells. *NRP2* KO consistently inhibited RENCA cell metabolic activity (Fig. S2B). Except for one *NRP1* KO clone (see discussion), *NRPs*’ KO decreased the migration velocity of 786-O cells which is consistent with the results obtained by down-regulating NRP1 and NRP2 (Fig. 1f and Fig. S1C). Since the NRPs’ signaling depends on stimulation by their ligands VEGFA and VEGFC, we tested their expression in the KO cells. In the 786-O model, *NRP1* KO and *NRP2* KO resulted in increased expression of their major ligands VEGFA and VEGFC respectively (Fig. 1g). Inconsistent increase in VEGFC (*NRP1* KO) or VEGFA (*NRP2* KO) has to be paralleled with variable up- or down-regulation of NRP2 or NRP1 which reflects a clonal specificity (Fig. 1c-d). In the RENCA model, expression of VEGFA was consistently decreased in *NRP1* and *NRP2* KO cells and VEGFC was down-regulated only in *NRP2* KO cells (Fig. S2C). Table S3 recapitulates VEGFA and VEGFC in the different model cell lines. These results suggest the maintenance of a steady state level of autocrine loops involving the respective NRP1/VEGFA and NRP2/VEGFC signaling pathways. This equilibrium varies from a model to another and is compensated, at least in the human model, by increased expression of the NRPs’ ligands.

### NRPs KO in tumor cells inhibited experimental RCC growth in immunocompetent and immunodeficient mice

**Fig. 2:**
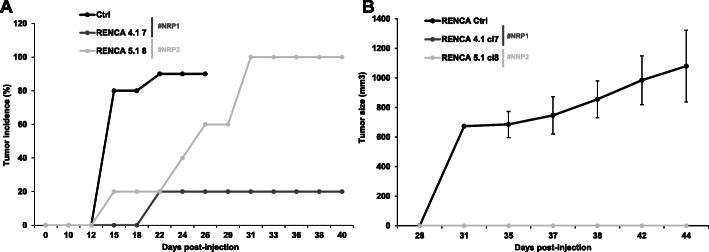
NRPs KO inhibits experimental RCC growth in immunocompetent and immunodeficient mice. **a** Experimental tumors in nude mice were obtained after injection of 3 × 10^5^ control (Ctrl, 10 mice) or NRPs KO RENCA cells (5 mice for each condition). One *NRP1* KO clone (4.1 7) and one *NRP2* KO clone (5.1 8) were injected. Tumor incidence (percentage of mice with tumors) at the indicated times is presented. **b** Experimental tumors in immuno-competent Balb-C mice (5 mice per condition) were obtained after subcutaneous injection of 3 × 10^5^ control (Ctrl) or the above-mentioned *NRPs* KO RENCA cells. The tumor volume is represented for the indicated time

Considering the relevance of immune checkpoint inhibitors in the treatment of mccRCC [26], we deciphered the specific role of NRPs expressed by tumor cells on the growth of experimental tumors in immunodeficient and immunocompetent mice. For that purpose, we compared the growth of experimental tumors generated with 786-O KO cells in nude mice and generated by RENCA KO cells grafted in nude and syngenic BalbC mice. The *NRP1* and *NRP2* 786-O KO clones generated smaller tumors in nude mice as compared to the controls (Fig. S3). This result is consistent with the NRPs-dependent cell metabolic activity in-vitro. Invalidation of *NRP1* or *NRP2* in RENCA cells delayed tumor incidence (percentage of mice with a tumor) as compared to the control group, in nude mice (Fig. 2a). Although, *NRP* KO cells generated tumors in some nude mice, their volumes remained very small as compared to control tumors (Fig. S4). Moreover, injection of the same cells in immunocompetent mice did not generate tumors (Fig. 2b). These results strongly suggest that, in addition to the intrinsic effects of NRPs on tumor cell metabolic activity, their expression on tumor cells inhibits the anti-tumor immune system.

### The NRP inhibitor NRPa-308 inhibits ccRCC cell metabolic activity more efficiently than sunitinib and EG00229

**Fig. 3:**
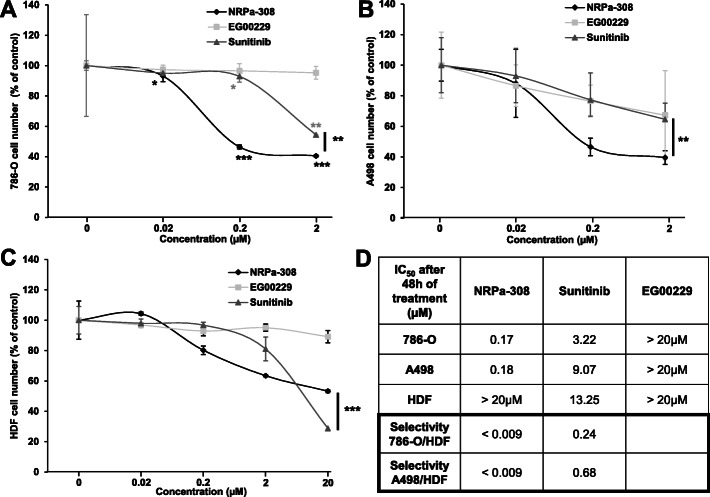
The NRP inhibitor NRPa-308 inhibits ccRCC cell proliferation more efficiently than sunitinib and EG00229. The effects of NRPa-308, sunitinib and the commercially available NRP inhibitor (EG00229) measured by MTT assays, were tested in (**a**) 786-O cells, (**b**) on A498 cells and (**c**) on HDF cells. d Determination of the IC_50_ for each treatment in the different cell lines and their selectivity index. *p < 0.05; **p < 0.01; *** *p* < 0.001

The strong impact on tumor growth mediated by invalidation of *NRPs* encouraged us to test the relevance of the NRPs’ pharmacological inhibitor NRPa-308 on different parameters characterizing ccRCC cells (786-O and A498) aggressiveness in comparison to its effect on normal dermal fibroblasts (HDF). The NRPa-308 effects were compared to those of the reference treatment for ccRCC, sunitinib, and to the commercially available NRPs’ inhibitor EG00229. EG00229 was poorly efficient in inhibiting the metabolic activity of ccRCC cell lines (5 and 30% inhibition respectively for 786-O and A498 cells at the highest dose 2 μM, Fig. 3a-b). Sunitinib inhibited ccRCC cell metabolic activity more efficiently especially in 786-O cells as compared to A498 cells (40% versus 30% for the highest dose). IC_50_ values were lower for NRPa-308 as compared to sunitinib suggesting its higher efficacy on cell metabolic activity. Moreover, the IC_50_ of NRPa-308 was higher in normal cells (HDF) as compared to tumor cells (Fig. 3c). We then calculated the selectivity index (SI) to evaluate the toxicity on normal tissues. The IC_50_ of normal cells (HDF) served as the reference value. The SI was below 1 which indicates that NRPa-308 is more efficient on tumor cells and that its general toxicity is low. The SI of NRPa-308 was below those of sunitinib suggesting a higher efficacy and a lower toxicity of NRPa-308 (Fig. 3d).

### NRPa-308 exerts a wide range anti-metabolic activity on primary ccRCC cells

**Fig. 4:**
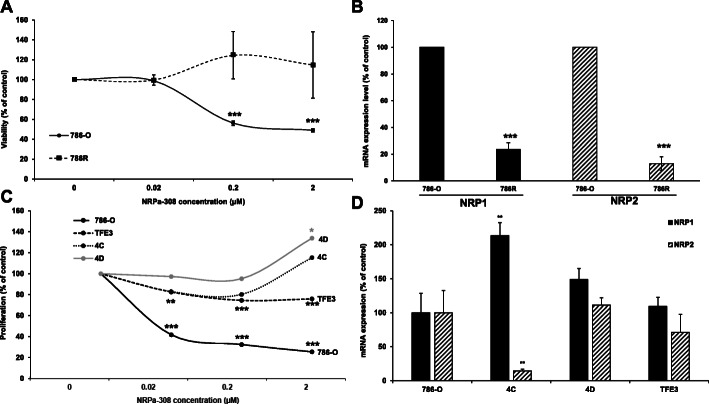
NRPa-308 exerts a wide range of anti-proliferative effect on primary ccRCC cells. **a** The effects of NRPa-308 on cell viability were tested on 786-O (786-O) and 786-O cells resistant to sunitinib (786R). **b** The relative expression of NRP1 and NRP2 mRNA in 786-O and 786R cells was evaluated in a RNA seq analysis and confirmed by RT qPCR. **c** The sensitivity of NRPa-308 was tested on already described primary cells [15] by MTT assays. **d** The relative mRNA levels were evaluated in 786-O cells that served as reference values (100%) and in the different primary cells

Resistance to the current treatments especially to sunitinib is a real concern [7]. We previously generated sunitinib-resistant 786-O cells (786R) by chronic exposure to the drug [27]. NRPa-308 had no effect on these cells (IC_50_ > 2 μM) as compared to the parental cells. 786R cells presented a four-fold and a nine-fold reduction of the NRP1 and NRP2 mRNA levels (Fig. 4a-b). These results are compatible with a strong dependence on cell proliferation/survival mediated by the VEGFA/NRP1 and VEGFC/NRP2 autocrine loops. We previously described primary ccRCC cells obtained from surgically operated tumor specimens and normal epithelial cells from the same donor [15]. Tumor cells presented a wide range of sensitivity to NRPa-308 (from 40 to 0% inhibition) as compared to normal primary kidney cells and to 786-O cells (Fig. 4c). TFE3 cells do not express VEGFC. Consequently, the NRP2/VEGFC pathway is not active in these cells. 4C cells express VEGFC equivalently to 786-O cells but do not express NRP2 and 4D cells express NRP2 but not VEGFC. In all the primary cells, the levels of VEGFA are modest and below to those of 786-O cells, hence limiting the activity of the NRP1/VEGFA pathway. These results suggest that the expression of NRPs and of their ligands VEGFA/VEGFC should be determined before the utilization of NRPa-308 in the clinic. Indeed, a difference in NRP1/VEGFA and/or NRP2/VEGFC expression seem(s) to influence NRPa-308 efficacy (Fig. 4d).

### NRPa-308-dependent inhibition of cell metabolic activity relied mainly on NRP2 in 786-O cells

**Fig. 5:**
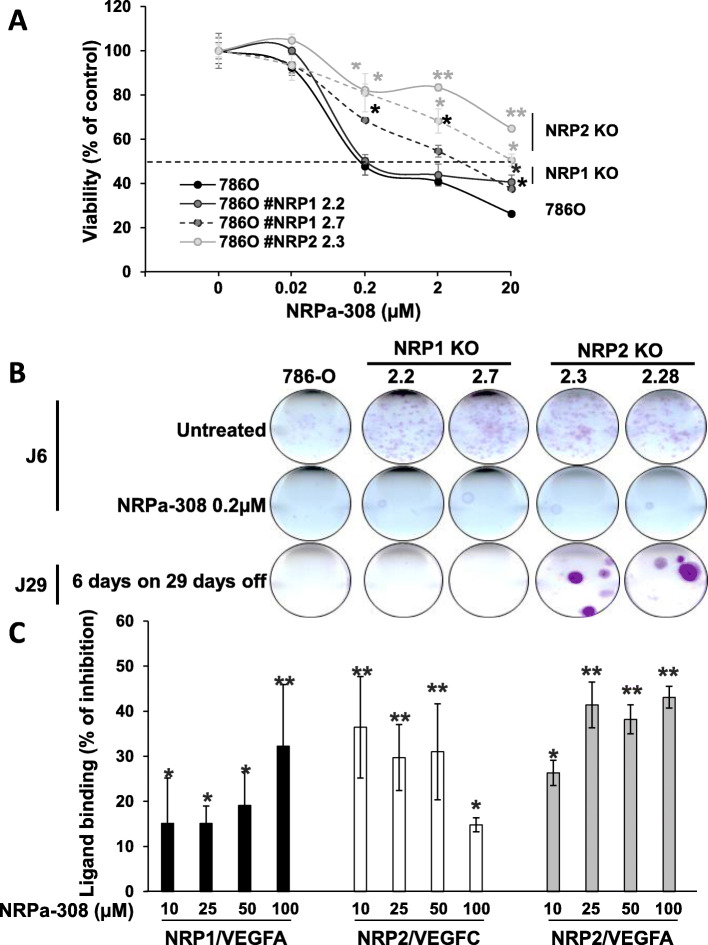
NRPa-308-dependent inhibition of cell proliferation relies mainly on NRP2 in 786-O cells. **a** Effects of NRPa-308 on cell viability of 786-O cells, of two independent *NRP1* (#NRP1 2.2 and #NRP1 2.7) KO clones and of two independent NRP2 (#NRP2 2.3 and #NRP2 2.28) KO clones, measured by MTT assays, are represented to determine NRPa-308 specificity to NRP1 and/or to NRP2. **b** Clonogenic tests were performed on control 786-O, NRP1- and NRP2-KO cells in the presence of NRPa-308 (0.2 μM) for 6 days or following 6 days treatment then a 29 days’ period without NRPa-308 (0.2 μM). **c** The percentage of inhibition of VEGFA binding to NRP1 and NRP2 and of VEGFC binding to NRP2 at different concentration of NRPa-308 is presented. *p < 0.05; **p < 0.01

*NRP1* and *NRP2* KO cells constitute ideal tools to test the specificity and the NRPs- dependent effect on cell metabolic activity of NRPa-308. NRPa-308 was designed to inhibit VEGFA binding on NRP1. VEGFA and VEGFC can interact with NRP1 and NRP2 and, as described above, VEGFA/NRP1 and VEGFC/NRP2 stimulate autocrine loops in ccRCC cells. Hence, we determined the specific anti-metabolic activity of NRPa-308 in NRPs’ KO clones. After 48 h of treatment, the IC_50_ of NRPa-308 was increased in each clone. This result strongly suggests that NRPa-308 exerts its anti-metabolic activity via NRP1 and NRP2. However, the increase in IC_50_ was higher for the *NRP2* KO clones, which suggest that NRPa-308 exerts its effects mainly via NRP2 (Fig. 5a). Clonogenic tests were also performed to further confirm the NRP2-dependency. No clone at all developed if NRPa-308 was removed in the culture medium of control or NRP1-KO cells. However, some clones emerged following treatment arrest of NRP2 KO clones which strongly suggests that NRPa-308 exerts its effects mainly via NRP2 (Fig. 5b). Affinity tests have been carried out to determine the efficacy of NRPa-308 to inhibit the VEGFA binding on NRP1 or NRP2 and the VEGFC binding on NRP2. The maximal inhibition reached approximately 40%. NRPa-308 inhibited VEGFA/NRP1 and VEGFA/NRP2 binding in a dose-dependent manner but surprisingly, inhibited VEGFC/NRP2 binding in a reverse dose-dependent manner. Hence, low doses of NRPa-308 are sufficient to prevent VEGFC binding to NRP2 which also suggests a stronger affinity for NRP2 as compared to NRP1 (Fig. 5c).

### NRPa-308 binding mode is different between NRP1 and NRP2

**Fig. 6:**
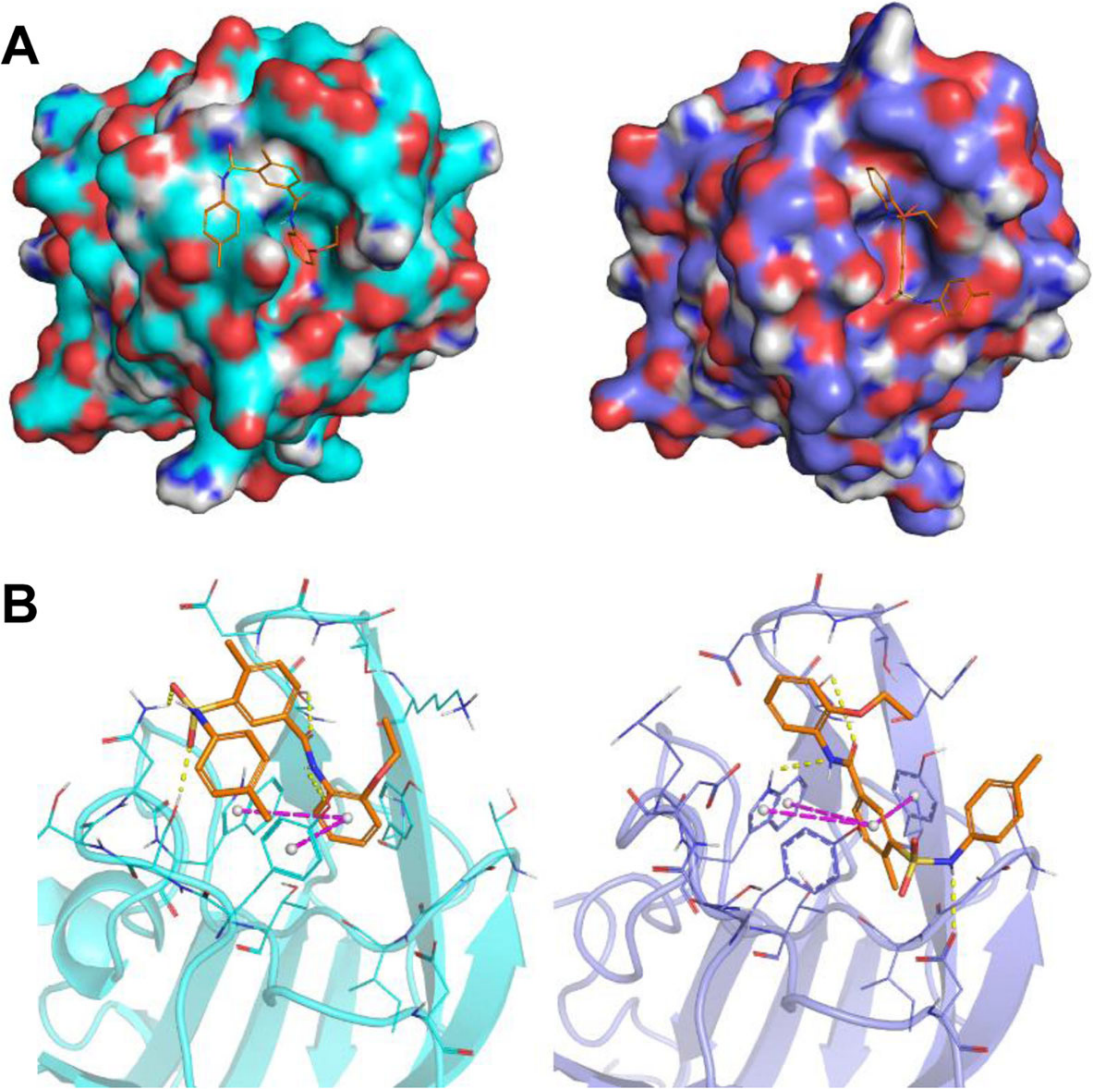
NRPa-308 binding mode is different between NRP1 and NRP2. NRPa-308 (colored in orange) predicted binding mode into the NRP1 (**a** and **b** in cyan, left panels) and NRP2 (**a** and **b** in blue, right panels) binding sites. Hydrogen bonds are depicted as yellow dashed lines and π-stacking are depicted as magenta dashed lines

To understand the mechanisms linked to the inhibition of VEGFA/VEGFC binding to NRP1 and NRP2, we conducted a docking study. The NRPa-308 predicted binding mode completely differs between NRP1 and NRP2 (Fig. 6a). The orientation of NRPa-308 into NRP1 binding site is flipped relatively to those obtained into the NRP2 binding site. In both cases, NRPa-308 is stabilized in the binding site through hydrogen bonds, π-stacking and hydrophobic interactions (Fig. 6b), but most of the interacting residues are distinct. Few residues involved in these interactions are conserved in NRP1 and NRP2 (W301/304, S346/349, E348/351, Y353/356 according to the NRP1/NRP2 numeration) but they establish interactions with different parts of NRPa-308. Comparison of the NRP1 and NRP2 structures revealed that the residues forming each binding site differ and consequently the NRP2 binding site is larger and more open than the NRP1 binding site. This result explains the docking study obtained with NRPa-308 but also the difference of affinity experimentally obtained (Fig. 5c).

### NRPa-308 inhibited 786-O cell migration and invasion

**Fig. 7:**
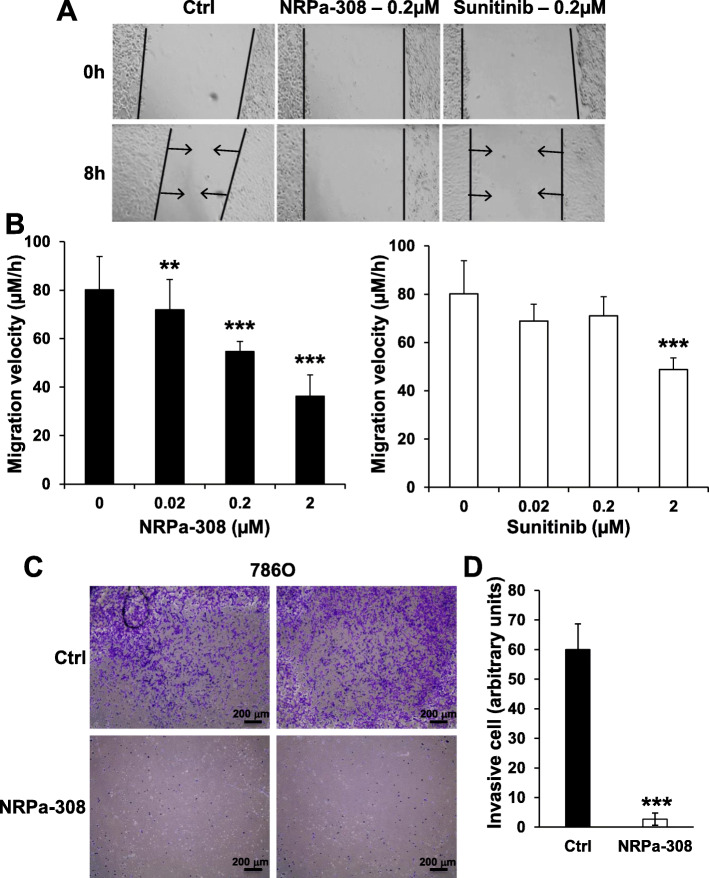
NRPa-308 inhibits 786-O cell migration velocity more efficiently than sunitinib. **a** Photographs of scratch assay on cell monolayers in different experimental conditions; untreated, treated by NRPa-308 and by sunitinib. **b** The effects of NRPa-308 and sunitinib on 786-O cell migration at different concentrations by quantifying the above-mentioned experiments. **c** NRPa-308 (2 μM) inhibited the invasion of collagen-coated Boyden chambers (Control (Ctrl) and NRPa-308-treated duplicates are shown). **d** Quantification of the results shown in (**c**). **p < 0.01; *** *p* < 0.001

As described above, NRPs down-regulation and KO resulted in the inhibition of cell migration. Therefore, the ability of NRPa-308 to inhibit this parameter of tumor cell aggressiveness was tested. NRPa-308 reduced 786-O cell migration more efficiently than sunitinib at a very low concentration (0.02 μM compared to 2 μM for sunitinib, Fig. 7a-b). NRPa-308 prevented also the ability of 786-O cells to invade a collagen matrix in a transwell system (Fig. 7c). This result suggests an anti-metastatic activity of NRPa-308.

### High NRPa-308 concentration stimulated the production of NRPs’ ligands and of pro-angiogenic/pro-inflammatory cytokines

**Fig. 8:**
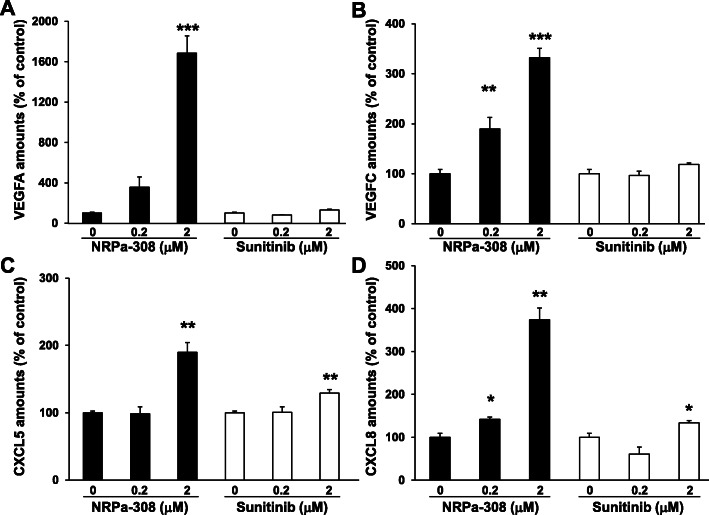
High NRPa-308 concentration stimulates the production of NRPs ligands and of pro-angiogenic/pro-inflammatory cytokines. The effects of NRPa-308 and sunitinib on the production of different cytokines were evaluated by ELISA; **a** VEGFA, **b** VEGFC, production (**c**) CXCL8, **d** CXCL5. *p < 0.05; **p < 0.01; *** *p* < 0.001

We observed that NRPs KO resulted in increased production of their ligands (Fig. 1). Although these ligands cannot influence tumor cells KO for NRPs, their paracrine effects can be highly detrimental by stimulating angio/lymphangiogenesis and by inducing immunotolerance. Hence, we evaluated the minimal NRPa-308 concentration, which inhibits cell metabolic activity without influencing their secretome. 0.2 μM of NRPa-308 maximally decreased the percentage of metabolically active cells (Fig. 3). Increasing further the concentration did not result in a better efficacy of the drug. Hence, we analyzed the expression of VEGFA and VEGFC following NRPa-308 treatment. We also determined the expression of the ELR + CXCL cytokines CXCL5 and CXCL8 since they are involved in resistance to bevacizumab and sunitinib in ccRCC as we previously described [16, 28]. Sunitinib, at these low concentrations (below the IC_50_ [27]), had no influence on VEGFA and VEGFC expression but increased CXCL5 and CXCL8 expression as previously shown (Fig. 8a-d) [28]. NRPa-308 increased the expression of these different factors at the highest concentration (2 μM). The lowest concentration (0.2 μM), only stimulated the expression of VEGFC. These results strongly suggest that the best ratio (beneficial/detrimental effects) can be obtained at low doses of NRPa-308 in the context of ccRCC.

### NRPa-308 decreased experimental ccRCC growth in a reverse dose-dependent manner

**Fig. 9:**
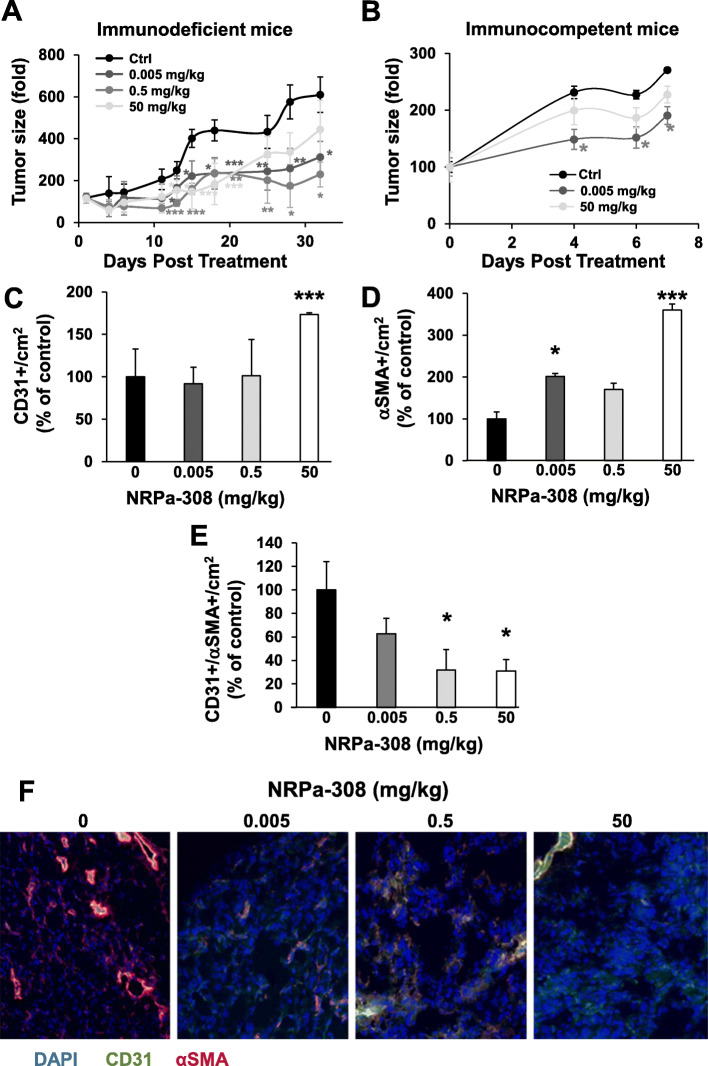
NRPa-308 decreases experimental ccRCC growth in a reverse dose-dependent manner. **a** Experimental tumors in nude mice (5 mice per condition) were obtained after injection of 3 × 10^6^ 786-O cells. Three concentrations of NRPa-308 (5 μg/kg, 500 μg/kg and 50 mg/kg) diluted in carboxymethyl cellulose, were given trice a week by oral gavage. The control group (Ctrl) received carboxymethyl cellulose. Tumor volume represented as a fold increase from the beginning of the treatment is presented. **b** Experimental tumors in immunocompetent mice (Balb-C, 5 mice per condition) were obtained after injection of 3 × 10^5^ RENCA cells. Treatment (NRPa-308) was given trice a week by oral gavage. Two concentrations of NRPa-308- (5 μg/kg and 50 mg/kg) diluted in carboxymethyl cellulose were administered. The control group (Ctrl) received carboxymethyl cellulose. Tumor volume represented as the fold increase from the beginning of the treatment is shown. The tumor vasculature in each experimental group was detected by immuno-staining for CD31 (endothelial cells, green) and α-SMA (pericytes, cancer associated fibroblasts), red). Tumor sections were counterstained with 40,6-diamidino-2-phenylindole (DAPI) (nucleus, blue). **c** Quantification of the blood vessels (CD31 labeling). **d** Quantification of pericytes and cancer associated fibroblasts (α-SMA). **e** Quantification of blood vessels covered with pericytes (yellow labeling). **f** Representative images of the dose-dependent decrease in blood vessels covered with pericytes. *p < 0.05; **p < 0.01; *** *p* < 0.001

In previous studies [8], NRPa-308 inhibited the growth of experimental breast cancers at an optimal dose of 50 mg/kg. A pilot experiment on experimental ccRCC generated in nude mice with 786-O cells was unsuccessful (no inhibition of tumor growth). The results presented in Fig. 8 suggested detrimental paracrine effects induced by high concentrations of NRPa-308. Therefore, we tested the effects of increasing concentrations of the drug (5 μg/kg, 500 μg/kg and 50 mg/kg) on the growth of experimental ccRCC in immunodeficient (xenograft of human 786-O cells) and immunocompetent (graft of syngenic mouse RENCA cells) mice. Considering a full distribution in the blood and a 1.5 ml of blood in a mouse of 25 g, the respective blood concentrations of the drug administered at 5 μg/kg, 500 μg/kg and 50 mg/kg should be around 0.2, 20 and 2000 μmol/L. Of course, these blood concentrations correspond to a rough estimation which does not consider the biological distribution in the organs especially metabolism of the drug in the liver. The lowest concentration is in the range of concentrations inhibiting cell metabolic activity, migration and invasion and impacting to a low extent the production of pro-angio/lymphangiogenic and pro-inflammatory cytokines. The highest NRPa-308 dose did not affect the growth of experimental ccRCC. However, tumor growth was inhibited significantly by the lower amounts, especially the lowest concentration of 5 μg/kg in both mouse models (Fig. 9a-b). Immunostaining were carried out on tumors generated in immunodeficient mice. The number of blood vessels (CD31 labeling) and of pericytes/cancer associated fibroblast (CAF, αSMA labeling) per cm^2^ was high in the control group and increased in a dose-dependent manner (Fig. 9c-d). However, the number of arterioles (CD31/αSMA co-labeling) decreased in a dose-dependent manner (Fig. 9e-f). Lymphatic vessels in these tumors were almost undetectable and NRPa-308 did not modify their number. These experiments showed that NRPa-308 represents an interesting therapeutic strategy for ccRCC at a low concentration which is a good compromise associating efficacy and low toxicity (no modification of mouse weight at low doses, Fig. S5).

### Efficient NRPa-308 dose decreased the expression of pro-tumoral factors

**Fig. 10:**
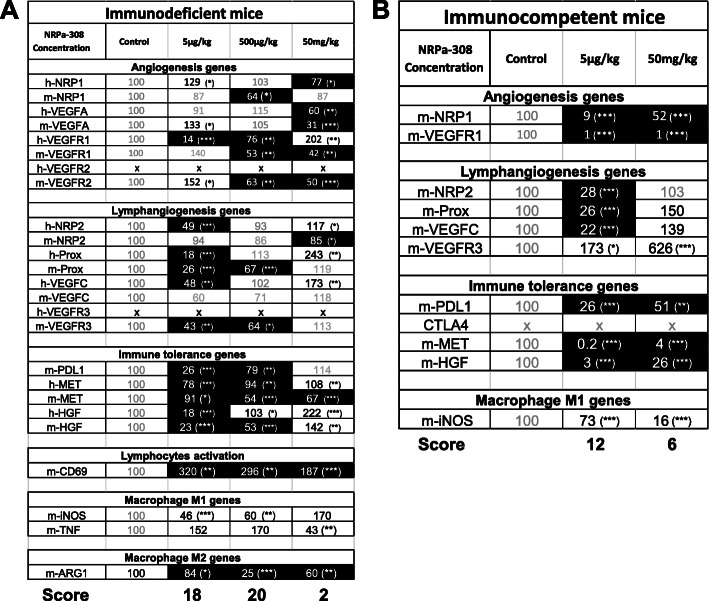
Efficient NRPa-308 dose decreases the expression of pro-tumoral factors. Detection by qPCR of pro-tumoral genes in tumors generated in immunodeficient and immunocompetent mice with wild-type and with NRPs knock-out cells. *p < 0.05; **p < 0.01; *** *p* < 0.001

To understand the better efficacy of low doses of NRPa-308, we evaluated the expression of genes involved in tumor aggressiveness especially those regulating proliferation, angio/lymphangiogenesis, epithelial/mesenchymal transition (EMT) and immune tolerance. The modifications to their mRNAs, analyzed by qPCR, were compiled in Fig. 10. Genes associated with lymphangiogenesis were the most downregulated by the lowest dose of the drug including human NRP2, Prox1 and VEGFC and murine Prox1 and VEGFC in the immunodeficient model (Fig. 10a) and NRP2, Prox1 and VEGFC in the immunocompetent model (Fig. 10b). Only murine Prox1 and VEGFC were downregulated by the intermediate dose in the immunodeficient model (Fig. 10a). Human NRP2, Prox1, and VEGFC and murine NRP2 were upregulated in the presence of the highest dose in the immunodeficient model (Fig. 10a). In the immunodeficient model, proangiogenic genes including human NRP1, VEGFA and murine NRP1, VEGFA, VEGFR1 and VEGFR2 were downregulated by the highest dose (Fig. 10a). Some of them were also downregulated by the lowest or intermediate dose including human VEGFA and VEGFR1 and murine NRP1, VEGFR1 and VEGFR2 (Fig. 10a). Human NRP1 and VEGFR1, and murine VEGFA and VEGFR2 were upregulated by using the lowest or the highest dose (Fig. 10a). In the immunocompetent model, the proangiogenic genes NRP1 and VEGFR1 were downregulated for the two doses (Fig. 10b). The murine gene involved in immunotolerance, PDL1 was downregulated by the lowest and intermediate dose and was unchanged for the highest dose in immunodeficient mice (Fig. 10a). It was downregulated by the two doses in immunocompetent mice. In the immunodeficient model, genes involved in EMT including human MET and HGF and murine MET and HGF were downregulated by the lowest or intermediate dose (Fig. 10a). Only murine MET was downregulated by the highest dose and human MET and HGF and murine HGF were upregulated by the intermediate or highest dose (Fig. 10a). In the immunocompetent model MET and HGF were downregulated by the two doses (Fig. 10b). In the immunodeficient model, mCD69, a marker of the lymphocytes’ activation, is upregulated, which is synonymous of an activation of the immune response (Fig. 10a). The M2 macrophages marker mARG1 was decreased, which reflects a beneficial polarization of macrophages (Fig. 10a). According to these differences, we attempted to establish a score of good or bad prognosis depending on the up or downregulation of genes involved in tumor aggressiveness. This score will serve to understand why we did not observe a dose dependent effect of NRPa-308. We gave a score of 2 when a gene of poor prognosis decreased and a score of − 2 when it increased and vice versa for a gene of good prognosis. The global score for the lowest concentration was respectively 18 and 12 in the immunodeficient (Fig. 10a) and immunocompetent models (Fig. 10b). It was of 20 for the intermediate dose in the immunodeficient model (Fig. 10a) and of 2 and 6 respectively for the highest dose in the immunodeficient (Fig. 10a) and the immunocompetent models (Fig. 10b). This evaluation, in addition to the reduction of tumor growth consistently favored the notion that a low dose of NRPa-308 had the best therapeutic efficacy that was not counterbalanced by the expression of genes related to tumor aggressiveness.

### The NRP2 associated pathway is more relevant for the aggressiveness of mccRCC

**Fig. 11:**
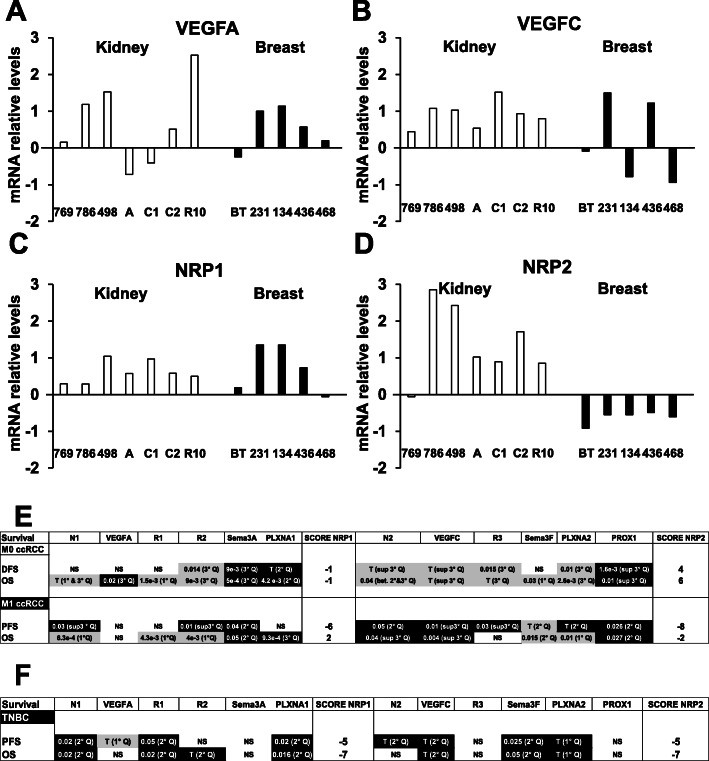
The NRP2 associated pathway is more determinant for the aggressiveness of mccRCC but not for triple negative breast cancers. Analysis of cbioportal database highlighted the relative levels of VEGFA (**a**), VEGFC (**b**), NRP1 (**c**) and NRP2 (**d**) mRNA in a panel of RCC (769 (769P), 786-O (786), ACHN (A), Caki1 (C1), Caki2 (C2), RCC10 (R10)) and TNBC (BT474 (BT), MDAMB231 (231), MDAMB134 (134), MDAMB436 (436), MDAMB468 (468)). Correlation between genes of the NRP1 and NRP2 pathways and survival (DFS/PFS/OS) in M0 and M1 RCC patients (**e**) and TNBC (**f**) patients. The tested genes of the NRP1 pathway were the following: *NRP1* (N1), *VEGFA*, *VEGFR1* (R1), *VEGFR2* (R2), *Semaphorin 3A* (Sema3A), *Plexin A1* (PLXNA1). The tested genes of the NRP2 pathway were the following: *NRP2* (N1), *VEGFC*, *VEGFR3* (R3), *Semaphorin 3F* (Sema3F), *Plexin A2* (PLXNA1) and *PROX1*. The *p*-values of genes associated with shorter DFS/PFS/OS appear white on a black background; the p-values of genes associated with a longer DFS/PFS/OS appear black on a gray background. Significant p-values are given; a trend to significance is indicated by a “T”. Specific cut-off are indicated (First, second or third quartile (1°, 2°, 3° Q). A score was established as follows: a positive point was given for a gene with a trend to good prognosis; two positive points for a gene associated with good prognosis and with a significant p-value; a negative point was given for a gene with a trend to poor prognosis; two negative points were given for a gene associated with poor prognosis and with a significant p-value. Positive scores were obtained for DFS and OS of M0 RCC patients and the NRP2 pathway (respectively 4 and 6) and for the OS of M1 RCC patients and the NRP1 pathway (2). Negative scores were obtained for obtained for the DFS and OS of M0 and PFS of M1 RCC patients and the NRP1 pathway (respectively (− 1), (− 1) (− 6), for the PFS and OS of M1 RCC patients and the NRP2 pathway. Negative score were obtained for the NRP1 and NRP2 pathways for PFS and OS

We were puzzled by confronting our previous results on breast cancers with those enclosed in this manuscript; high dose of NRPa-308 was the most efficient on models of breast cancers whereas it has no effect in models of ccRCC [8]. Considering the striking therapeutic value of targeting NRPs for both models of cancers, we first analyzed the relative expression of NRPs and their ligands VEGFA and VEGFC on a panel of cell lines available in the TCGA data base. In most of the cell lines representative of aggressive ccRCC and breast cancers, VEGFA and NRP1 are expressed at high levels especially in the cell lines used in our respective experimental tumor growth (786-O and MDAMB231) (Fig. 11a-c). VEGFC and NRP2 are expressed by all the ccRCC cell lines. However, VEGFC levels are very low in three out of five breast cancer cell lines and NRP2 levels are very low in all the breast cell lines including MDAMB231 (Fig. 11b-d). These very low levels in MDAMB231 and the more specific effects of NRPa-308 on NRP2, partly explained the results obtained on experimental tumor growth. Our next step was to deep insight into the prognostic role of NRP1 and NRP2 and their known partners VEGFA, VEGFR1, VEGFR2, Semaphorin 3A (Sema3A) and plexin A1 (PLXNA1) (all NRP1 partners) and VEGFC, VEGFR3, Semaphorin 3F (Sema3F), plexin A2 (PLXNA1) and Prospero homeobox protein 1 (Prox1), a master transcription factor of lymphangiogenesis (all NRP2 partners). For that purpose, we correlated the expression of these different partners to disease free survival (DFS, non-metastatic patients M0), progression free survival (PFS, metastatic patients M1) and overall survival (OS) in patients with ccRCC and in patients with the most severe triple negative breast cancers (TNBC). For each gene, we defined the best cut off that determines a survival difference. Four hundred twenty-five samples were from M0 and 103 from M1 ccRCC patients. One hundred fifteen samples were from TNBC patients.

For M0 ccRCC patients, expression of VEGFR2, NRP2, VEGFC, VEGFR3, PLXA2 above their respective best cut off was of good prognosis for DFS (trend (T, p between 0.08 and 0.06) for NRP2, VEGFC) and significant (S) for VEGFR2, VEGFR3, PLXA2). Expression above the best cut off for Sema3A and Prox1 was of poor prognosis for DFS. For M0 patients OS, NRP1, VEGFR1, VEGFR2, NRP2, VEGFC, VEGFR3, Sema3F and PLXA2 (T for NRP1, VEGFC, VEGFR3 S for VEGFR1, VEGFR2, NRP2 and Sema3F). For M1 ccRCC patients, only Sema3F (T) was correlated to a longer PFS whereas eight parameters were correlated to a worse prognosis (NRP1 (S), VEGFR2 (S), Sema3A (S), NRP2 (S), VEGFC (S), VEGFR (S)3, PLXA2 (T) and Prox1 (S)). NRP1 (S), VEGFR1 (S), VEGFR2 (S), Sema3F (S) and PLXA2 (S) were correlated with a longer OS while Sema3A (S), PLXA1 (S), NRP2 (S), VEGFC (S) and Prox1 (S) were correlated to a shorter one.

For TNBC, VEGFA was curiously associated with a longer PFS (T) but NRP1 (S), VEGFR1 (S), PLXNA1 (S), NRP2 (T), VEGFC (T), Sema3F (S) and PLXNA2 (T) were correlated with a shorter one. NRP1 (S), VEGFR1 (S), VEGFR2 (T), PLXNA1 (S), VEGFC (T), Sema3F (S) and PLXNA2 (T) were correlated with a shorter OS. NRP2 was not correlated to survival in that case.

We defined a score by attributing a relative weight of − 2 for a gene associated with a significant poor prognosis and a relative weight of − 1 for a trend. Inversely, a relative weight of 2 was given for a gene associated with a significant good prognosis and 1 for a trend. NRP1 and NRP2 pathways were considered separately. For the NRP1 pathway, a − 1 score was obtained for the DFS and OS of M0 ccRCC patients, − 6 and 2 scores for PFS and OS of M1 ccRCC patients, and − 5 and − 7 scores for the PFS and OS of TNBC patients. For the NRP2 pathway, positive score of 4 and 6 were obtained for the DFS and OS of ccRCC patients, − 8 and − 2 scores for M1 ccRCC patients and − 5 and − 7 scores for the PFS and OS of TNBC patients.

These results showed that NRP1 and NRP2 signaling pathways, in general, strongly correlate with shorter survival for the most aggressive cancers, M1 ccRCC and TNBC. However, NRP2 is correlated with shorter DFS and OS in ccRCC while NRP1 is more involved in TNBC patients’ survival. These results suggest that NRP1 targeting is more adapted for TNBC while NRP2 targeting is more adapted for ccRCC.

## Discussion

NRPs, through their direct effect on tumor cells (stimulation of metabolic activity and migration/invasion) and on cells of the microenvironment (angio/lymphangiogenesis and immune tolerance) are key signaling molecules stimulating ccRCC growth and metastasis. However, the multi partnerships of NRPs render difficult the determination of the relative importance of each pathway. Moreover, we discovered that NRP1 and NRP2 signaling cross-talked to establish a steady state depending on the production of VEGFA and VEGFC. This phenomenon was strikingly observed in NRP1 KO clones in which compensatory expression of NRP2 was insufficient to inhibit cell migration (Fig. 1f)*.* We also showed that inflammatory cytokines compensate for the inhibition of NRPs’ pathways. These compensatory mechanisms are key for an optimized targeting of NRPs in the context of ccRCC treatment. Our results had to be compared to those of Cao Y et al who showed that inhibition of experimental tumor growth generated with cells downregulated for NRPs only relies on microenvironment shaping [10, 29]. By generating the same models, we showed discrepant results at longer time points as compared to those of Cao Y et al. These discrepancies depend on the stimulation of alternative autocrine pathways mediated by a modified secretome. However, modifications of these secretomes also depend on a partial or complete inhibition by KO of the NRPs’ signaling. These compensatory mechanisms are particularly striking if NRPs’ signaling inhibition enters in a therapeutic strategy. We are aware that KO or pharmacological inhibition may induce different responses. However, it was the only way to generate compelling evidence for demonstrating the relevance of targeting both NRPs for an optimal therapeutic strategy. The puzzling results that we obtained in the present and in our previous study, highlight the relative importance of NRP1 or NRP2 signaling depending on the cancer type. NRPa-308, discovered by its ability to inhibit VEGFA binding to NRP1 [13], is a better NRP2 inhibitor. Analysis of the TCGA database revealed that NRP1 is a better therapeutic target for TNBC and NRP2 is a better one for ccRCC. This result suggests that NRP1 or NRP2 inhibitors are more relevant for a specific cancer. The importance of the double KO was questioned in 786-O cells. Several attempts were unsuccessful suggesting that the double KO is lethal. Hence, an inhibitor of NRP1/VEGFA or NRP2/VEGFC is relevant but must not induce compensatory signaling pathways as for conventional anti-angiogenic drugs [30]. Therefore, an inhibitor of both NRP1 and NRP2 will be more efficient.

Anti-angiogenic drugs, immunotherapies or the combo are the current standard of care [31, 32]. Our results suggest that resistance to anti-angiogenics especially sunitinib, involved a down-regulation of NRPs. Therefore, NRPs’ inhibitors do not seem relevant at relapse on sunitinib. However, NRPs’ inhibitors represent an alternative following failure of immunotherapies used in the first line treatment of M1 patients [14]. Adjuvant treatment for M0 ccRCC patients is a debated issue. While some trials showed that an adjuvant treatment by anti-angiogenics is not relevant, another trial demonstrated its importance for advanced M0 patients [33, 34]. Our results showed that the NRP2 pathway is correlated with a good prognosis for M0 patients and NRP1 did not correlate with shorter survival rates. Our results emphasized the relevance of NRPs targeting in only M1 ccRCC patients and anti NRPs should not be used in an adjuvant setting.

## Conclusions

Comparison of ccRCC and TNBC highlighted the relevance of the NRP1 and the NRP2 pathways. Predetermination of NRPs’ expression is important to administrate NRP inhibitors. Although NRPa-308 represents an interesting hit if tumor cells express both NRPs and their ligands VEGFA/VEGFC, specific drugs targeting NRP1 should be more appropriate if only NRP1/VEGFA is present. This concept must be further addressed in depth to reach the “golden age” of the therapeutic arsenal for ccRCC [35].

## Supplementary Information

**Additional file 1: Table S1.** List of oligonucleotides used in qPCR experiments. **Table S2.** Recapitulative table of the expression of NRP1 and NRP2 in KO and knock-down cells. **Table S3.** Recapitulative table of the expression of VEGFA and VEGFC in KO and knock-down cells. **Table S4**. NRP1 and NRP2 binding site descriptors computed with DogSite Scorer (Nb: number, HBA: Hydrogen Bond Acceptor, HBD: Hydrogen Bond Donor, AA: amino acids).

**Additional file 2: Fig. S1.** Study of down-regulation of NRPs by shRNA in 786-O cells. (A-B) Effects of the downregulation of NRP by shRNA on *NRP1* and *NRP2* mRNA expression measured by qPCR. (C) Effects on cell metabolic activity measured by MTT assays. (D) Down-regulation of NRPs decreased cell migration. Bevacizumab increased this effect for NRP1 down-regulation. (E) Down-regulation of NRPs had no effect on VEGFA and VEGFC production measured by ELISA. *p < 0.05; **p < 0.01; *** *p* < 0.001.

**Additional file 3: Fig. S2.** Effects of *NRP1* or *NRP2* gene invalidation in RENCA cells. (A) NRP1 and NRP2 protein levels were evaluated by flow cytometry in control (RENCA), in #NRP1 4.1.7 and #NRP2 5.1.8 clones*.* (B) Effects of NRPs KO on RENCA cell metabolic activity measured by MTT assays. (C) Effects of NRPs KO in RENCA cells on the VEGFA and VEGFC protein levels measured by ELISA. *p < 0.05; **p < 0.01; *** p < 0.001.

**Additional file 4: Fig. S3.** NRPs KO in 786-O tumor cells inhibited experimental RCC growth in immunodeficient mice. (A) Experimental tumors in nude mice (5 mice per condition) were obtained after injection of 3 × 10^6^ wildtype (Ctrl) or NRPs KO 786-O cells. One NRP1 (#NRP1 2.7) clone and one NRP2 (#NRP2 2.3) clone were injected. Tumor volume is presented. *p < 0.05; **p < 0.01; *** *p* < 0.001.

**Additional file 5: Fig. S4.** NRPs KO in RENCA tumor cells inhibited experimental RCC growth in immunodeficient mice. (A) Experimental tumors in nude mice were obtained after injection of 3 × 10^5^ control (Ctrl, 10 mice) or NRPs KO RENCA cells (5 mice for each condition). One *NRP1* KO clone (4.1 7) and one *NRP2* KO clone (5.1 8) were injected. Tumor volume at the indicated times is presented. Each curve stands for an individual mouse.

**Additional file 6: Fig. S5.** In-vivo effects of NRPa-308 on mice weight. The weight of nude mice xenografted with 786-O cells and treated with increasing doses of NRPa-308 was evaluated once a week.

## Data Availability

All data generated or analyzed during this study are included in this published article and its supplementary information files. Patient datasets are those of the TCGA and are publicly available.

## Abbreviations

ATCC: American type culture collection
BSA: Bovine serum albumin
ccRCC: Clear cell Renal Cell Carcinoma
CXCL: C-X-C motif chemokine
DNA: Deoxyribonucleic acid
EMT: Epithelial/mesenchymal transition
GFP: Green fluorescent protein
HDF: Normal dermal fibroblast
HGF: Hepatocyte growth factor
HIF: Hypoxia inducible factor
HRP: Horseradish peroxidase
IC_50_: Half maximal inhibitory concentration
KO: Knock-out
Luc: Luciferase
mccRCC: Metastatic clear cell Renal Cell Carcinoma
MET: c-MET tyrosine-kinase receptor
mRNA: Messenger ribonucleic acid
NRP: Neuropilin
PBS: Phosphate buffered saline
PEI: Polyethylenimine
qPCR: Quantitative polymerase chain reaction
sgRNA: Single guide ribonucleic acid
shRNA: Short-hairpin ribonucleic acid
SI: Selectivity index
TBS: Tris-buffered saline
TCGA: The cancer genome atlas
TKi: Tyrosine-kinase inhibitor
VEGF: Vascular endothelial growth factor
VEGFR: Vascular endothelial growth factor receptor
VHL: Von-Hippel Lindau
αSMA: α smooth muscle actin
