# Supplementary material for: Neuropilin 1 and Neuropilin 2 gene invalidation or pharmacological inhibition reveals their relevance for the treatment of metastatic renal cell carcinoma
Source: J Exp Clin Cancer Res. 2021 Jan 18;40:33. doi: 10.1186/s13046-021-01832-x (PMC7812727; doi:10.1186/s13046-021-01832-x)
Supplement: Supplementary file 1 — Additional file 1: Table S1. List of oligonucleotides used in qPCR experiments. Table S2. Recapitulative table of the expression of NRP1 and NRP2 in KO and knock-down cells. Table S3. Recapitulative table of the expression of VEGFA and VEGFC in KO and knock-down cells. Table S4. NRP1 and NRP2 binding site descriptors computed with DogSite Scorer (Nb: number, HBA: Hydrogen Bond Acceptor, HBD: Hydrogen Bond Donor, AA: amino acids). [file 13046_2021_1832_MOESM1_ESM.docx]

**Supplemental methods**

**Downregulation by shRNA**

The targeting sequence for NRP1 were 5’- TGTGGATGACATTAGTATTAA -3’ (shNRP1); 5’-TACTGTGCCTGTTGGCATAAT-3’ (shNRP1bis) and 5’- CCTCAACTTCAACCCTCACTT -3’ (shNRP2); 5’-CGTTTCCAGATGACAGGAATT-3’ (shNRP2bis). These shRNA (mission shRNA Sigma/Merck) were expressed in cells through lentiviral infection.

**Supplemental Tables**

|  | **Forward (5' to 3')** | **Reverse (5' to 3')** |
| --- | --- | --- |
| 36B4 | CAGATTGGCTACCCAACTGTT | GGCCAGGACTCGTTTGTACC |
| m-RPLP0 | AGATTCGGGATATGCTGTTGGC | TCGGGTCCTAGACCAGTGTTC |
| GAPDH | TGCACCACCAACTGCTTAGC | GGCATGGACTGTGGTCATGAG |
|  |  |  |
| **Angiogenesis genes** | | |
| h-NRP1 | GGCGCTTTTCGCAACGATAA | TCGCATTTTTTCACTTGGGTGAT |
| m-NRP1 | GACAAATGTGGCGGGACCATA | TGGATTAGCCATTCACACTTCTC |
| h-VEGF-A | TTTCTGCTGTCTTGGGTGCATTGG | ACCACTTCGTGATGATTCTGCCCT |
| m-VEGFA | GCACATAGAGAGAATGAGCTTCC | CTCCGCTCTGAACAAGGCT |
| h-VEGFR1 | ATGGAAAACGCATAATCTGCA | AAATGCCCATTGACTGTTGCT |
| m-VEGFR1 | TGGCTCTAC GACCTTAGACTG | CAGGTTTGACTTGTCTGAGGTT |
| h-VEGFR2 | CATGTTGGTCACTAACAGAAG | GTGATCGGAAATGACACTGGA |
| m-VEGFR2 | TTTGGCAAATACAACCCTTCAGA | GCAGAAGATACTGTCACCACC |
|  |  |  |
| **Lymphangiogenesis genes** | | |
| h-NRP-2 | GCTGGCTATATCACCTCTCCC | TCTCGATTTCAAAGTGAGGGTTG |
| m-NRP-2 | GCTGGCTACATCACTTCCCC | CAATCCACTCACAGTTCTGGTG |
| h-Prox | AGTTCAACAGATGCATTACC | TCTCTGGTTATAGACAGCTC |
| m-Prox | AGAAGGGTTGACATTGGAGTGA | TGCGTGTTGCACCACAGAATA |
| h-VEGFC | TTACGGTCTGTGTCCAGTGTA | TTCTCTGTTATGTTGCCAGCC |
| m-VEGF-C | CTCTGTGGGACCACATGGTAA | TCCTCTCCCGCAGTAATCCA |
| h-VEGFR3 | TGCACGAGGTACATGCCAAC | GCTGCTCAAAGTCTCTCACGAA |
| m-VEGFR3 | CGAGTCGGAGCCTTCTGAGG | GCAGTCCAGCAATAGGGGGT |
|  |  |  |
| **Immune tolerance genes** | | |
| m-PDL1 | CCAGGATGGTTCTTAGACTCCC | TTTAGCACGAAGCTCTCCGAT |
| h-MET | AGCGTCAACAGAGGGACCT | GCAGTGAACCTCCGACTGTATG |
| m-MET | AGCGTCAACAGAGGGACCT | GCAGTGAACCTCCGACTGTATG |
| h-HGF | GCTATCGGGGTAAAGACCTACA | CGTAGCGTACCTCTGGATTGC |
| m-HGF | ATGTGGGGGACCAAACTTCTG | GGATGGCGACATGAAGCAG |
|  |  |  |
| **Lymphocyte activation** | | |
| m-CD69 | AAAAGGACATGACGTTTCTG | CAGCTGTTAAATTCTTTGCC |
|  |  |  |
| **Macrophage M1 genes** | | |
| m-iNOS | TCACCTTCGAGGGCAGCCGA | TCCGTGGCAAAGCGAGCCAG |
| m-TNF | CTATGTCAGCCTCTTCTC | CATTTGGGAACTTCTCATCC |
|  |  |  |
| **Macrophages M2 genes** | | |
| m-ARG1 | GATTATCGGAGCGCCTTTCT | CCACACTGACTCTTCCATTCTT |

**Table S1. List of oligonucleotides used in qPCR experiments**

|  |  | NRP1 | NRP2 |
| --- | --- | --- | --- |
|  |  |  |  |
|  | KO NRP1 | --- | +++ |
| **786-O** |  |  |  |
|  | KO NRP2 | - | --- |
|  |  |  |  |
|  | KO NRP1 | --- | --- |
| **RENCA** |  |  |  |
|  | KO NRP2 | - | --- |
|  |  |  |  |
|  | SH NRP1 | -- | - |
| **786-O** |  |  |  |
|  | SH NRP2 | - | -- |

**Table S2. Recapitulative table of the expression of NRP1 and NRP2 in KO and knock-down cells.** Three (-) mean a statistically significant decrease, three (+) mean a statistically significant increase. A (-) appearing on a grey background means a trend toward a decreased expression of the examined gene.

|  |  | VEGFA | VEGFC |
| --- | --- | --- | --- |
|  |  |  |  |
|  | KO NRP1 | +++ | + |
| **786-O** |  |  |  |
|  | KO NRP2 | - | +++ |
|  |  |  |  |
|  | KO NRP1 | --- | - |
| **RENCA** |  |  |  |
|  | KO NRP2 | --- | --- |
|  |  |  |  |
|  | SH NRP1 | - | - |
| **786-O** |  |  |  |
|  | SH NRP2 | + | - |

**Table S3. Recapitulative table of the expression of VEGFA and VEGFC in KO and knock-down cells.** Three (-) mean a statistically significant decrease, three (+) mean a statistically significant increase. A (-) appearing on a grey background means a trend toward a decreased expression of the examined gene. A (+) appearing on a grey background means a trend toward an increased expression of the examined gene.

|  | NRP1 (6FMF) | NRP2 (5DN2) |
| --- | --- | --- |
| Volume (Å^3^) | 317.632 | 284.096 |
| Surface (Å^2^) | 319.52 | 302.24 |
| Depth (Å) | 14.1421 | 14.1365 |
| Nb of HBA | 16 | 15 |
| Nb of HBD | 15 | 12 |
| Hydrophobicity | 0.707317 | 0.706667 |
| Nb of negative AA | 3 | 3 |
| Nb of positive AA | 1 | 1 |
| Nb of polar AA | 12 | 8 |
| Nb of apolar AA | 3 | 5 |
| Total nb of AA | 19 | 17 |

**Table S4**. NRP1 and NRP2 binding site descriptors computed with DogSite Scorer (Nb: number, HBA: Hydrogen Bond Acceptor, HBD: Hydrogen Bond Donor, AA: amino acids)
