# Supplementary material for: Neuropilin 1 and Neuropilin 2 gene invalidation or pharmacological inhibition reveals their relevance for the treatment of metastatic renal cell carcinoma
Source: J Exp Clin Cancer Res. 2021 Jan 18;40:33. doi: 10.1186/s13046-021-01832-x (PMC7812727; doi:10.1186/s13046-021-01832-x)
Supplement: Supplementary file 2 — Additional file 2: Fig. S1. Study of down-regulation of NRPs by shRNA in 786-O cells. (A-B) Effects of the downregulation of NRP by shRNA on NRP1 and NRP2 mRNA expression measured by qPCR. (C) Effects on cell metabolic activity measured by MTT assays. (D) Down-regulation of NRPs decreased cell migration. Bevacizumab increased this effect for NRP1 down-regulation. (E) Down-regulation of NRPs had no effect on VEGFA and VEGFC production measured by ELISA. *p < 0.05; **p < 0.01; *** p < 0.001. [file 13046_2021_1832_MOESM2_ESM.pptx]

## Slide 1
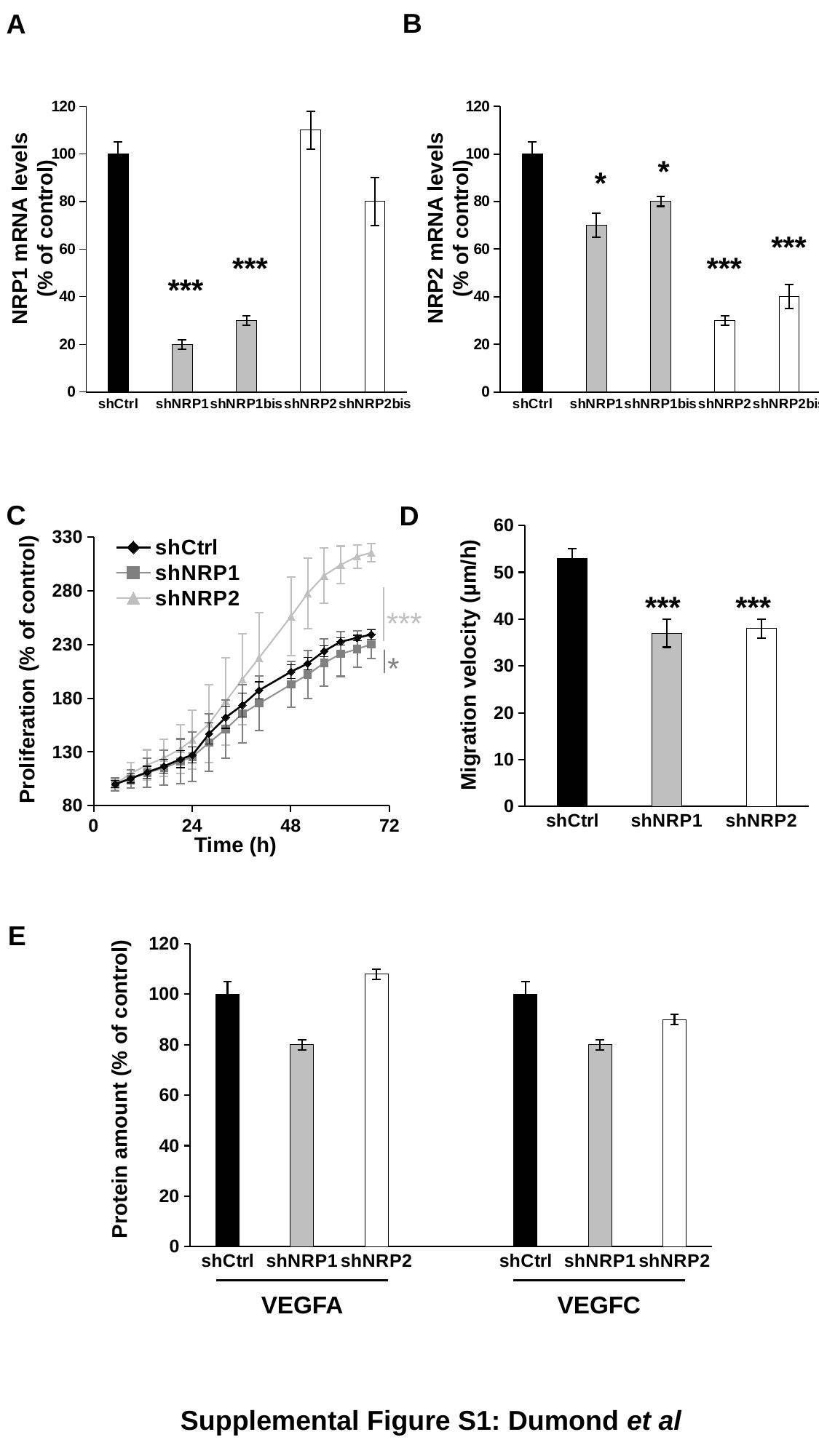

B
A
### Chart
| Category | |
|---|---|
| shCtrl | 100.0 |
| shNRP1 | 20.0 |
| shNRP1bis | 30.0 |
| shNRP2 | 110.0 |
| shNRP2bis | 80.0 |
### Chart
| Category | |
|---|---|
| shCtrl | 100.0 |
| shNRP1 | 70.0 |
| shNRP1bis | 80.0 |
| shNRP2 | 30.0 |
| shNRP2bis | 40.0 |*
*
NRP1 mRNA levels
(% of control)
NRP2 mRNA levels
(% of control)
***
***
***
***
C
D
### Chart
| Category | |
|---|---|
| shCtrl | 53.0 |
| shNRP1 | 37.0 |
| shNRP2 | 38.0 |***
***
Migration velocity (µm/h)
### Chart
| Category | shCtrl | shNRP1 | shNRP2 |
|---|---|---|---|***
*
Proliferation (% of control)
Time (h)
E
### Chart
| Category | |
|---|---|
| shCtrl | 100.0 |
| shNRP1 | 80.0 |
| shNRP2 | 108.0 |
| | None |
| shCtrl | 100.0 |
| shNRP1 | 80.0 |
| shNRP2 | 90.0 |Protein amount (% of control)
VEGFA
VEGFC
Supplemental Figure S1: Dumond et al
