# Supplementary material for: Neuropilin 1 and Neuropilin 2 gene invalidation or pharmacological inhibition reveals their relevance for the treatment of metastatic renal cell carcinoma
Source: J Exp Clin Cancer Res. 2021 Jan 18;40:33. doi: 10.1186/s13046-021-01832-x (PMC7812727; doi:10.1186/s13046-021-01832-x)
Supplement: Supplementary file 3 — Additional file 3: Fig. S2. Effects of NRP1 or NRP2 gene invalidation in RENCA cells. (A) NRP1 and NRP2 protein levels were evaluated by flow cytometry in control (RENCA), in #NRP1 4.1.7 and #NRP2 5.1.8 clones. (B) Effects of NRPs KO on RENCA cell metabolic activity measured by MTT assays. (C) Effects of NRPs KO in RENCA cells on the VEGFA and VEGFC protein levels measured by ELISA. *p < 0.05; **p < 0.01; *** p < 0.001. [file 13046_2021_1832_MOESM3_ESM.pptx]

## Slide 1
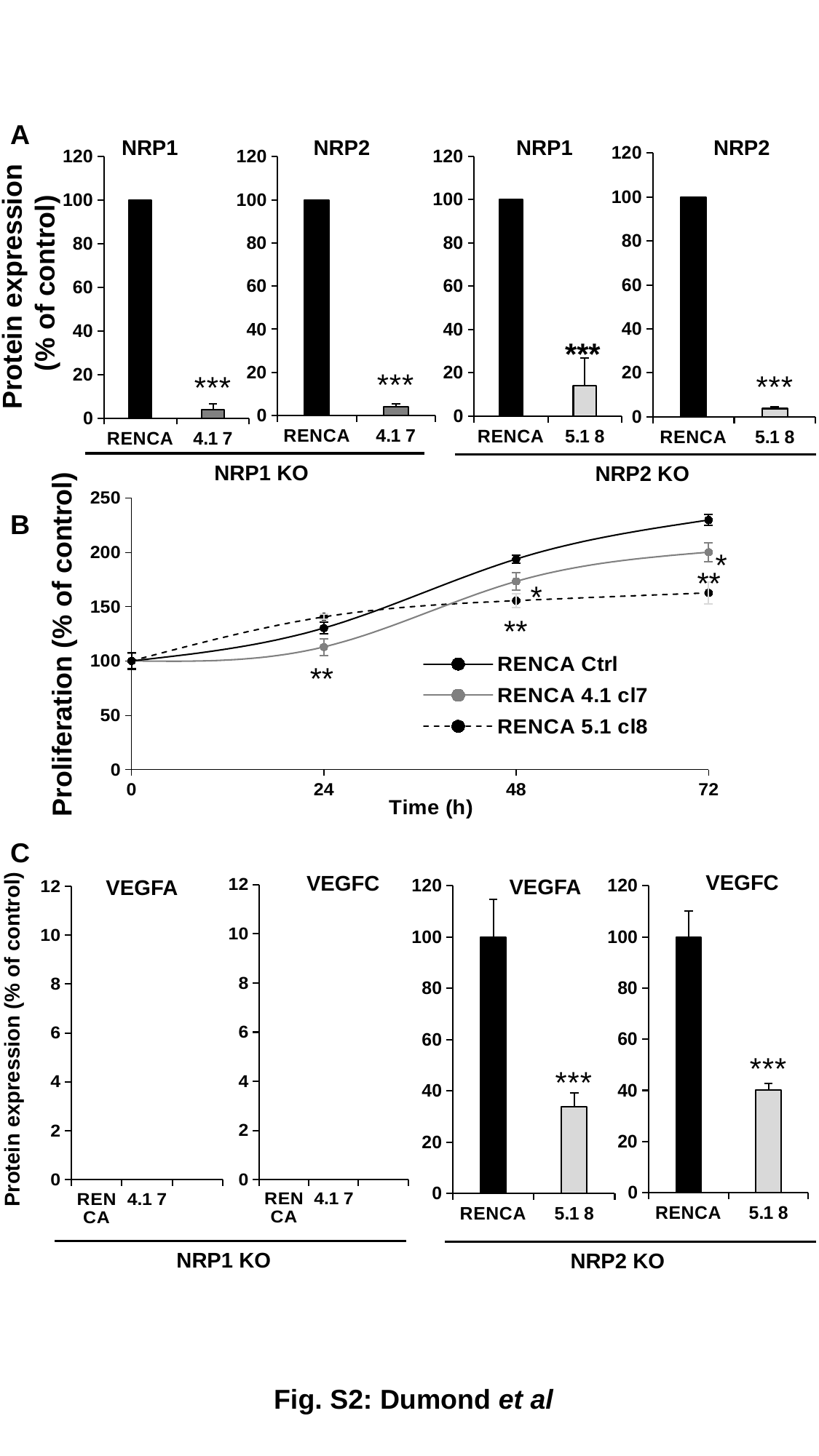

A
NRP1
NRP2
NRP1
NRP2
### Chart
| Category | |
|---|---|
| RENCA | 100.0 |
| 5.1 8 | 3.71 |
### Chart
| Category | |
|---|---|
| RENCA | 100.0 |
| 5.1 8 | 14.084999999999999 |
### Chart
| Category | NRP1 |
|---|---|
| RENCA | 100.0 |
| 4.1 7 | 3.94 |
### Chart
| Category | |
|---|---|
| RENCA | 100.0 |
| 4.1 7 | 4.085 |Protein expression
 (% of control)
***
NRP1 KO
NRP2 KO
### Chart
| Category | RENCA Ctrl | RENCA 4.1 cl7 | RENCA 5.1 cl8 |
|---|---|---|---|B
*
**
*
**
Proliferation (% of control)
**
C
VEGFC
VEGFC
VEGFA
VEGFA
### Chart
| Category | VEGFA |
|---|---|
| RENCA | 100.0 |
| 4.1 7 | 32.481838989821156 |
### Chart
| Category | |
|---|---|
| RENCA | 100.0 |
| 4.1 7 | 86.95114586645457 |
### Chart
| Category | VEGFA |
|---|---|
| RENCA | 100.0 |
| 5.1 8 | 33.906431867360666 |
### Chart
| Category | |
|---|---|
| RENCA | 100.0 |
| 5.1 8 | 40.07592580298455 |Protein expression (% of control)
NRP1 KO
NRP2 KO
Fig. S2: Dumond et al
