# Supplementary figures and images for: Neuropilin 1 and Neuropilin 2 gene invalidation or pharmacological inhibition reveals their relevance for the treatment of metastatic renal cell carcinoma
Source: J Exp Clin Cancer Res. 2021 Jan 18;40:33. doi: 10.1186/s13046-021-01832-x (PMC7812727; doi:10.1186/s13046-021-01832-x)

## Slide 1
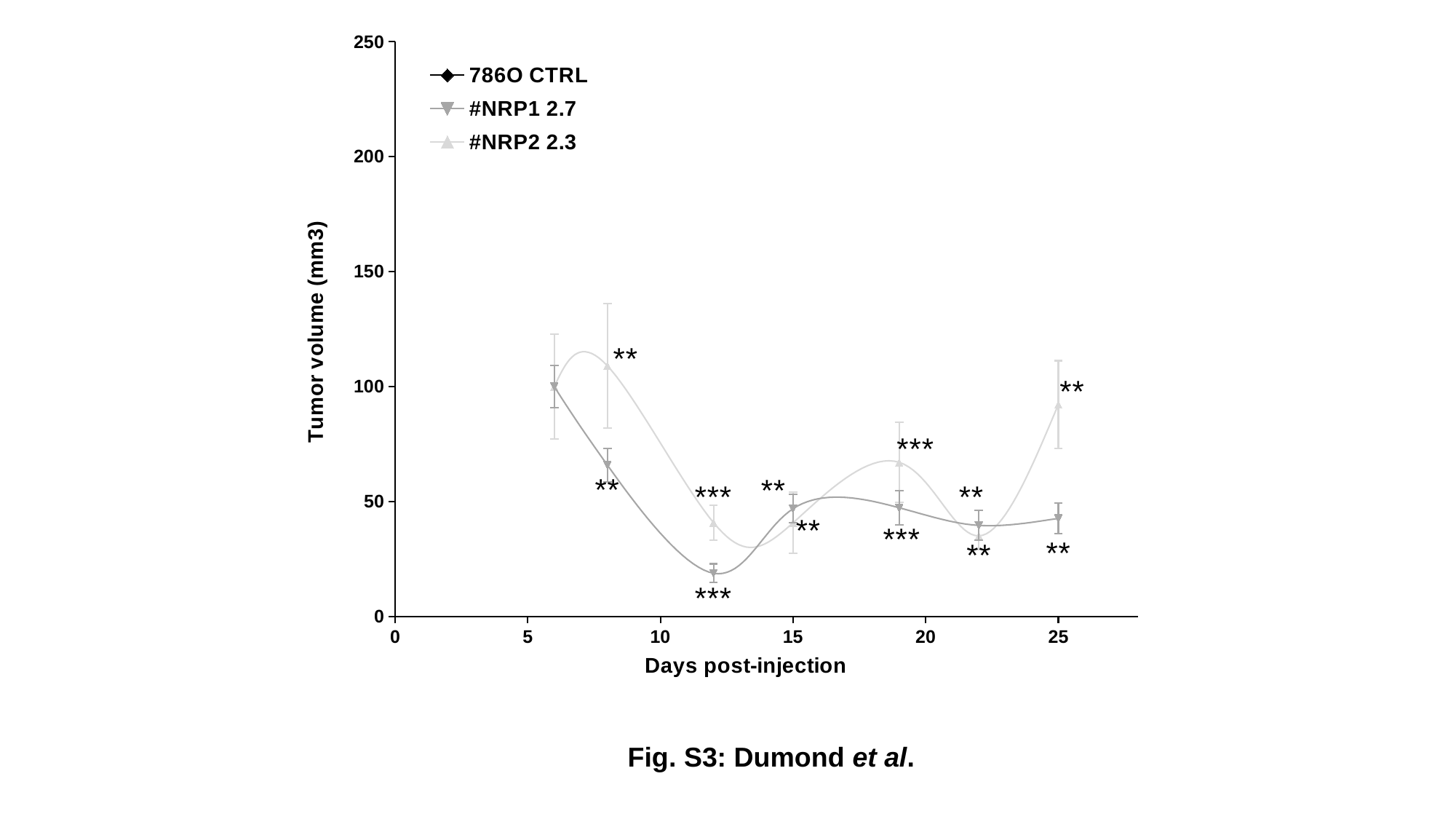

### Chart
| Category | 786O CTRL | #NRP1 2.7 | #NRP2 2.3 |
|---|---|---|---|Fig. S3: Dumond et al.

Supplement: Supplementary file 4 — Additional file 4: Fig. S3. NRPs KO in 786-O tumor cells inhibited experimental RCC growth in immunodeficient mice. (A) Experimental tumors in nude mice (5 mice per condition) were obtained after injection of 3 × 10^6^ wildtype (Ctrl) or NRPs KO 786-O cells. One NRP1 (#NRP1 2.7) clone and one NRP2 (#NRP2 2.3) clone were injected. Tumor volume is presented. *p < 0.05; **p < 0.01; *** p < 0.001. [file 13046_2021_1832_MOESM4_ESM.pptx]
