# Supplementary material for: Neuropilin 1 and Neuropilin 2 gene invalidation or pharmacological inhibition reveals their relevance for the treatment of metastatic renal cell carcinoma
Source: J Exp Clin Cancer Res. 2021 Jan 18;40:33. doi: 10.1186/s13046-021-01832-x (PMC7812727; doi:10.1186/s13046-021-01832-x)
Supplement: Supplementary file 5 — Additional file 5: Fig. S4. NRPs KO in RENCA tumor cells inhibited experimental RCC growth in immunodeficient mice. (A) Experimental tumors in nude mice were obtained after injection of 3 × 10^5^ control (Ctrl, 10 mice) or NRPs KO RENCA cells (5 mice for each condition). One NRP1 KO clone (4.1 7) and one NRP2 KO clone (5.1 8) were injected. Tumor volume at the indicated times is presented. Each curve stands for an individual mouse. [file 13046_2021_1832_MOESM5_ESM.pptx]

## Slide 1
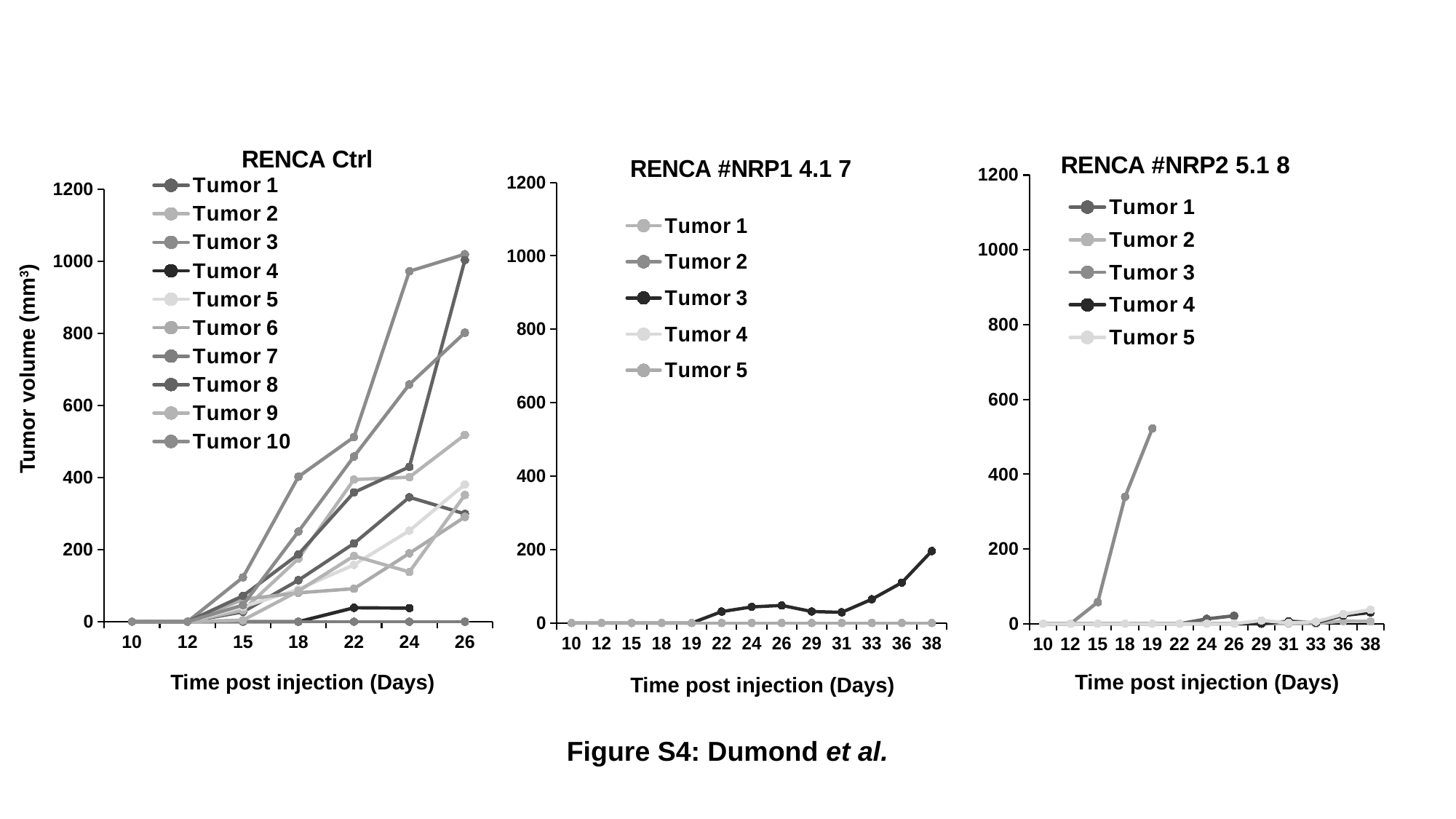

[unsupported chart]
### Chart: RENCA #NRP1 4.1 7
| Category | Tumor 1 | Tumor 2 | Tumor 3 | Tumor 4 | Tumor 5 |
|---|---|---|---|---|---|
| 10 | 0.0 | 0.0 | 0.0 | 0.0 | 0.0 |
| 12 | 0.0 | 0.0 | 0.0 | 0.0 | 0.0 |
| 15 | 0.0 | 0.0 | 0.0 | 0.0 | 0.0 |
| 18 | 0.0 | 0.0 | 0.0 | 0.0 | 0.0 |
| 19 | 0.0 | 0.0 | 0.0 | 0.0 | 0.0 |
| 22 | 0.0 | 0.0 | 31.1 | 0.0 | 0.0 |
| 24 | 0.0 | 0.0 | 43.8 | 0.0 | 0.0 |
| 26 | 0.0 | 0.0 | 48.0 | 0.0 | 0.0 |
| 29 | 0.0 | 0.0 | 31.3 | 0.0 | 0.0 |
| 31 | 0.0 | 0.0 | 29.1 | 0.0 | 0.0 |
| 33 | 0.0 | 0.0 | 64.7 | 0.0 | 0.0 |
| 36 | 0.0 | 0.0 | 110.0 | 0.0 | 0.0 |
| 38 | 0.0 | 0.0 | 196.1 | 0.0 | 0.0 |
### Chart: RENCA Ctrl
| Category | Tumor 1 | Tumor 2 | Tumor 3 | Tumor 4 | Tumor 5 | Tumor 6 | Tumor 7 | Tumor 8 | Tumor 9 | Tumor 10 |
|---|---|---|---|---|---|---|---|---|---|---|
| 10 | 0.0 | 0.0 | 0.0 | 0.0 | 0.0 | 0.0 | 0.0 | 0.0 | 0.0 | 0.0 |
| 12 | 0.0 | 0.0 | 0.0 | 0.0 | 0.0 | 0.0 | 0.0 | 0.0 | 0.0 | 0.0 |
| 15 | 27.17600048 | 31.52941064 | 122.798551195 | 0.0 | 41.122823175 | 61.710919529999984 | 0.0 | 71.55463306600002 | 4.184 | 46.08667109000001 |
| 18 | 115.0 | 175.0 | 402.09757327600005 | 0.0 | 90.0 | 80.0 | 0.0 | 186.848979429 | 85.0 | 250.0 |
| 22 | 216.99058551100003 | 394.01449740000004 | 511.822649016 | 38.5913332 | 157.6163008 | 91.537973538 | 0.0 | 358.377932196 | 182.28328199999999 | 458.05601894399996 |
| 24 | 345.167957925 | 400.74061682700005 | 971.893061559 | 37.598039571 | 252.306143775 | 189.61371223700002 | 0.0 | 429.31536930100003 | 137.97320373099998 | 658.144558231 |
| 26 | 299.002286116 | 518.1164352 | 1019.1256178040003 | None | 380.7390838000001 | 290.61066482100006 | 0.0 | 1002.8646670720002 | 351.44789245400005 | 801.573368472 |Tumor volume (mm3)
Time post injection (Days)
Time post injection (Days)
Time post injection (Days)
Figure S4: Dumond et al.
