# Supplementary material for: Neuropilin 1 and Neuropilin 2 gene invalidation or pharmacological inhibition reveals their relevance for the treatment of metastatic renal cell carcinoma
Source: J Exp Clin Cancer Res. 2021 Jan 18;40:33. doi: 10.1186/s13046-021-01832-x (PMC7812727; doi:10.1186/s13046-021-01832-x)
Supplement: Supplementary file 6 — Additional file 6: Fig. S5. In-vivo effects of NRPa-308 on mice weight. The weight of nude mice xenografted with 786-O cells and treated with increasing doses of NRPa-308 was evaluated once a week. [file 13046_2021_1832_MOESM6_ESM.pptx]

## Slide 1
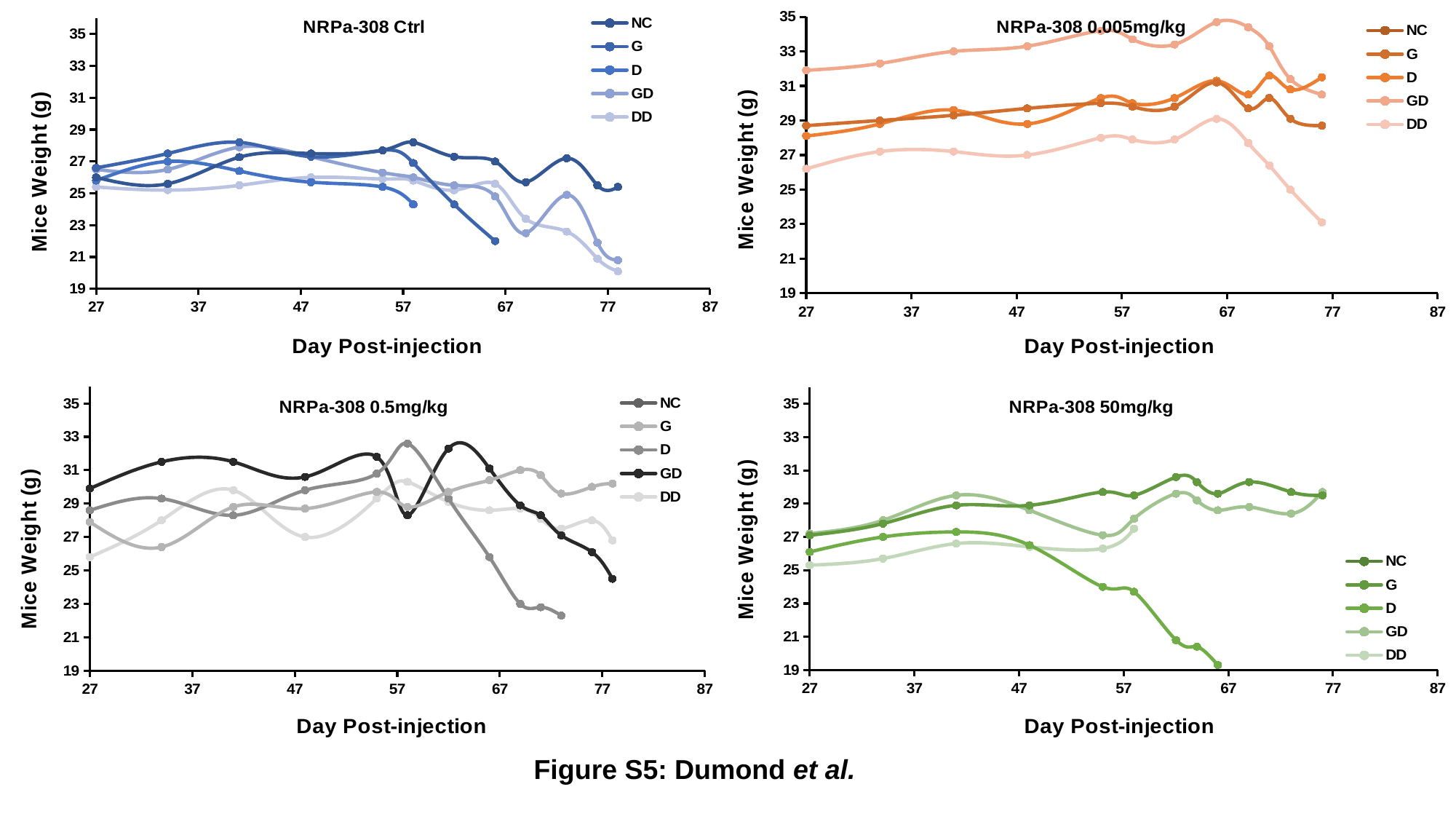

### Chart: NRPa-308 Ctrl
| Category | NC | G | D | GD | DD |
|---|---|---|---|---|---|
### Chart: NRPa-308 0.005mg/kg
| Category | NC | G | D | GD | DD |
|---|---|---|---|---|---|
### Chart: NRPa-308 0.5mg/kg
| Category | NC | G | D | GD | DD |
|---|---|---|---|---|---|
### Chart: NRPa-308 50mg/kg
| Category | NC | G | D | GD | DD |
|---|---|---|---|---|---|Figure S5: Dumond et al.
